# Sensory input, sex and function shape hypothalamic cell type development

**DOI:** 10.1038/s41586-025-08603-0

**Published:** 2025-03-05

**Authors:** Harris S. Kaplan, Brandon L. Logeman, Kai Zhang, Tate A. Yawitz, Celine Santiago, Noor Sohail, Mustafa Talay, Changwoo Seo, Serhiy Naumenko, Shannan J. Ho Sui, David D. Ginty, Bing Ren, Catherine Dulac

**Affiliations:** 1 Department of Molecular and Cellular Biology, Howard Hughes Medical Institute, Center for Brain Science, Harvard University, Cambridge, MA USA; 2 Department of Cellular and Molecular Medicine, Center for Epigenomics, University of California, San Diego School of Medicine, La Jolla, CA USA; 3 Department of Neurobiology, Harvard Medical School, Howard Hughes Medical Institute, Boston, MA USA; 4 Department of Biostatistics, Harvard Chan School of Public Health, Boston, MA USA; 5 Newborn Screening Ontario, Ottawa, Ontario Canada; 6 Present Address: Westlake Laboratory of Life Sciences and Biomedicine, School of Life Sciences, Westlake University, Hangzhou, China

**Keywords:** Cell type diversity, Social behaviour

## Abstract

Mammalian behaviour and physiology undergo major changes in early life. Young animals rely on conspecifics to meet their needs and start showing nutritional independence and sex-specific social interactions at weaning and puberty, respectively. How neuronal populations regulating homeostatic functions and social behaviours develop during these transitions remains unclear. We used paired transcriptomic and chromatin accessibility profiling to examine the developmental trajectories of neuronal populations in the hypothalamic preoptic region, where cell types with key roles in physiological and behavioural control have been identified^1–6^. These data show a marked diversity of developmental trajectories shaped by the sex of the animal, and the location and behavioural or physiological function of the corresponding cell types. We identify key stages of preoptic development, including early diversification, perinatal emergence of sex differences, postnatal maturation and refinement of signalling networks, and nonlinear transcriptional changes accelerating at the time of weaning and puberty. We assessed preoptic development in various sensory mutants and find a major role for vomeronasal sensing in the timing of preoptic cell type maturation. These results provide new insights into the development of neurons controlling homeostatic functions and social behaviours and lay ground for examining the dynamics of these functions in early life.

## Abstract (web summary)

Paired transcriptomic and chromatin accessibility profiling are used to examine the developmental trajectories of neuronal populations in the hypothalamic preoptic region, including cell types with key roles in physiological and social behaviour functions have been identified.

## Main

Animal behaviour changes considerably over postnatal development. Sensory experiences are altered as sensory organs mature, and homeostatic needs such as sleep, thermoregulation and hunger are met differently as young animals reach independence. Social relationships are also transformed; in mammals, interactions with parents change with weaning, and sex-specific reproductive and defensive behaviours emerge after puberty. How these changes affect, or are affected by, the maturation of neuronal circuits is mostly unknown. Our current understanding of neuronal circuit development mainly comes from studies of sensory and cortical pathways, in which the emergence, spatial organization, connectivity and activity patterns of distinct cell types involve interplays between genetic and activity-dependent mechanisms^7–10^. By contrast, despite fierce debates on the roles of genetic and environmental information in the emergence of species- and sex-specific behaviours^11–13^, developmental mechanisms underlying the corresponding brain circuits are poorly understood. Distinct physiological and social functions emerge at various stages of postnatal development, yet how behavioural and homeostatic transitions are reflected in the development of specific neuronal populations remains unclear. In addition, although environmental information is essential for the proper maturation of social and survival behaviours^14–16^, how critical periods may shape the corresponding neuronal populations is unknown^17^.

**Fig. 1:**
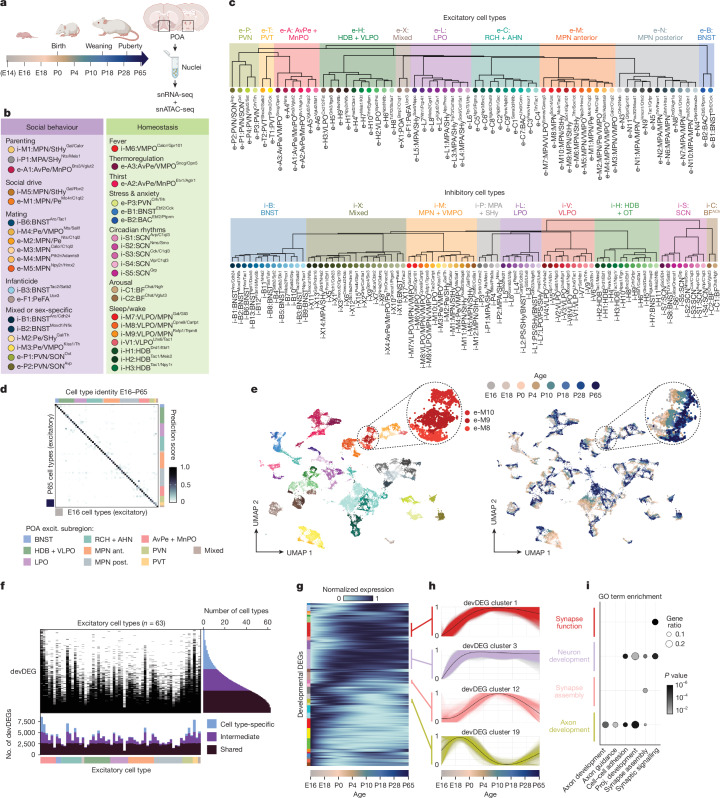
A molecular atlas of hypothalamic POA cell types across development. **a**, Schematic of experiments: POA and surrounding regions were dissected, nuclei extracted and snRNA-seq + snATAC-seq libraries prepared at eight developmental stages, two male and two female samples per age. E, embryonic; P, postnatal. **b**, Cell types with previously identified roles in social behaviour or homeostatic control. **c**, Hierarchical clustering of snRNA-seq clusters at P65, comprising 64 excitatory (top) and 83 inhibitory (bottom) clusters, organized according to subregional POA identity (shading on tree). ‘e-’ and ‘i-’ denote excitatory and inhibitory cell types, respectively, followed by a single letter indicating POA subregion. **d**, Mapping of E16 onto P65 clusters using canonical correlation analysis for excitatory neurons. Prediction score is averaged across cells within a cluster. Almost all E16 cell types map to a single P65 cell type, with very few off-diagonal correspondence. **e**, Uniform manifold approximation and projection for dimension reduction (UMAP) across all eight ages for excitatory clusters. Left, coloured by cluster (key in **c**, top); right, by age. Inset, gradient across age for three clusters. **f**, DEGs were determined within each cluster across all ages (devDEGs). Right histogram, devDEGs are categorized according to the number of clusters in which they are identified: more than 70% (‘shared’), less than 20% (‘cell type-specific’) or in between (‘intermediate’). Bottom histogram, number of devDEGs for each cluster. **g**, Normalized cell type-specific gene expression changes (fit with a trend curve) across all devDEGs, clustered (left coloured bars) according to time course. **h**, Four examples of devDEG clusters. Each coloured line indicates the cell type-specific gene expression change of a single devDEG; each black line indicates the mean across all devDEGs in the cluster. **i**, GO term enrichment for the four examples of devDEG clusters shown in **h**. ACh, acetylcholinergic; AHN, anterior hypothalamic nucleus; AvPe, anteroventral periventricular nucleus; BF, basal forebrain; BNST, bed nucleus of the stria terminalis; HDB, horizontal limb of the diagonal band nucleus; LPO, lateral preoptic area; MnPO, median preoptic nucleus; MPA, medial preoptic area; MPN, medial preoptic nucleus; OT, olfactory tubercle; PVN, paraventricular hypothalamic nucleus; PVT, paraventricular thalamic nucleus; RCH, retrochiasmatic area; SCN, suprachiasmatic nucleus; SHy, septohypothalamic nucleus; StHy, striohypothalamic nucleus; VLPO, ventrolateral preoptic nucleus; VMPO, ventromedial preoptic nucleus. Illustrations in **a** created using BioRender (https://biorender.com).

In vertebrates, the preoptic area (POA) of the hypothalamus is essential for homeostatic and social behaviour control. Molecularly defined cell types in specific POA subregions have been associated with various physiological and social functions in adult mice. For example, ventrolateral preoptic nucleus (VLPO) Tac1+ (ref. ^3^) and Gal+ (ref. ^5^) neurons are involved in sleep–wake control, median preoptic nucleus (MnPO) AGTR1A^+^ neurons in thirst^2^ and medial preoptic nucleus (MPN) Gal+ neurons in parenting^1,18,19^ (see the legend of Fig. 1 for all POA subregion acronyms). In early postnatal life, the coordinated regulation of these functions may be especially critical, as survival needs such as warmth and nutrient intake require interactions with conspecifics. How and when these different neuronal populations emerge and mature remains unexplored.

POA neurons are born between embryonic day 12 (E12) and E16 from progenitors lining the third ventricle and migrate radially to their ultimate location, with little to no migration into the POA from adjacent regions^20^. We use paired single-nucleus gene expression and chromatin accessibility sequencing (single-nucleus RNA sequencing (snRNA-seq) and single-nucleus assay for transposase-accessible chromatin using sequencing (snATAC-seq)) to uncover the developmental trajectories of 147 neuronal types in POA and surrounding areas in mice at several embryonic and postnatal stages from E14 to adult, mapping cell populations to a previously published atlas of adult POA cell types^1^. Cell type diversity emerged rapidly following neurogenesis, and cell type identity was apparent at E16 with further maturational trajectories varying according to POA subregion, behavioural function and sex. We delineated the emergence of signalling networks involved in social or homeostatic functions and identified key transcription factor drivers of these changes. Finally, by profiling the POA of various sensory mutants, we identified a major role for vomeronasal input in ensuring normal POA developmental timing. Our findings provide new insights into POA development and lay the foundation for a mechanistic understanding of critical periods in instinctive behaviour circuit development and function in early life.

## Transcriptional atlas of POA cell type development

We performed droplet-based paired snRNA-seq and snATAC-seq at eight ages from E16 to P65 that capture key transitions associated with birth, weaning and puberty in both male and female mice (Fig. 1a). After quality control filtering, we obtained snRNA-seq profiles from 206,413 nuclei and snATAC-seq profiles from 198,753 nuclei.

**Extended Data Fig. 1:**
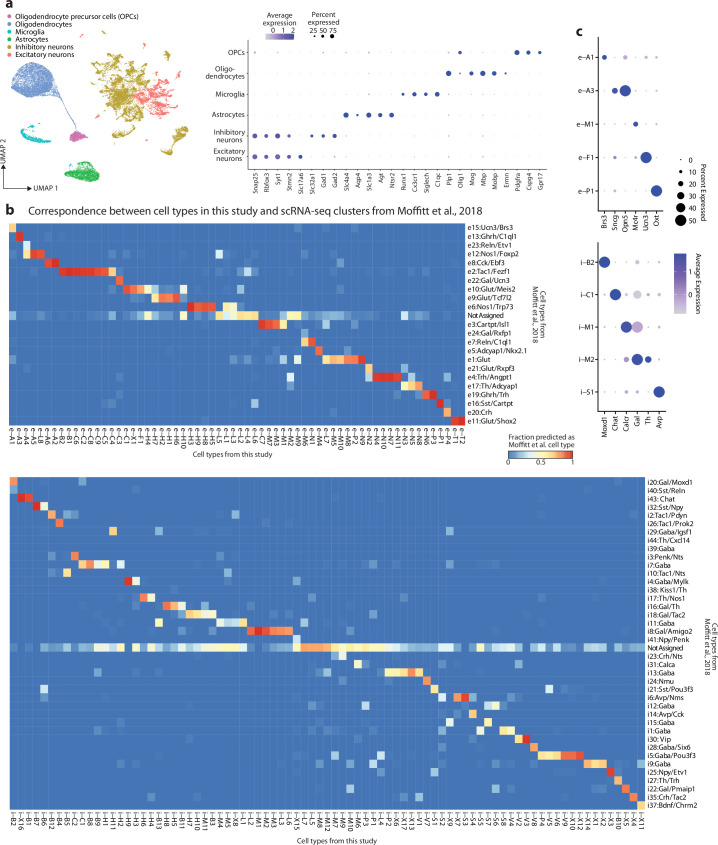
Identifying major cell classes and mapping cell types to reference. (**a**) UMAP colored by cell class (excitatory/inhibitory neurons, oligodendrocytes, astrocytes, microglia) and associated Dotplot with gene markers to verify class IDs. (**b**) Mapping of cell types in this study (columns) to scRNA-seq cell types in the POA reference atlas^1^ (Moffitt et al., 2018), for excitatory (upper) and inhibitory (lower) neurons. Canonical correlation analysis was used to generate a prediction score, which was then thresholded (Methods) to give predicted cell identity or NA for cells that did not pass threshold for any cell type. Color represents the fraction of cells in each cell type in this study that were predicted to belong to each reference atlas cell type. (**c**) Dotplot showing gene markers for previously studied cell types (see Fig. 1b for cell type function and full name).

**Extended Data Fig. 2:**
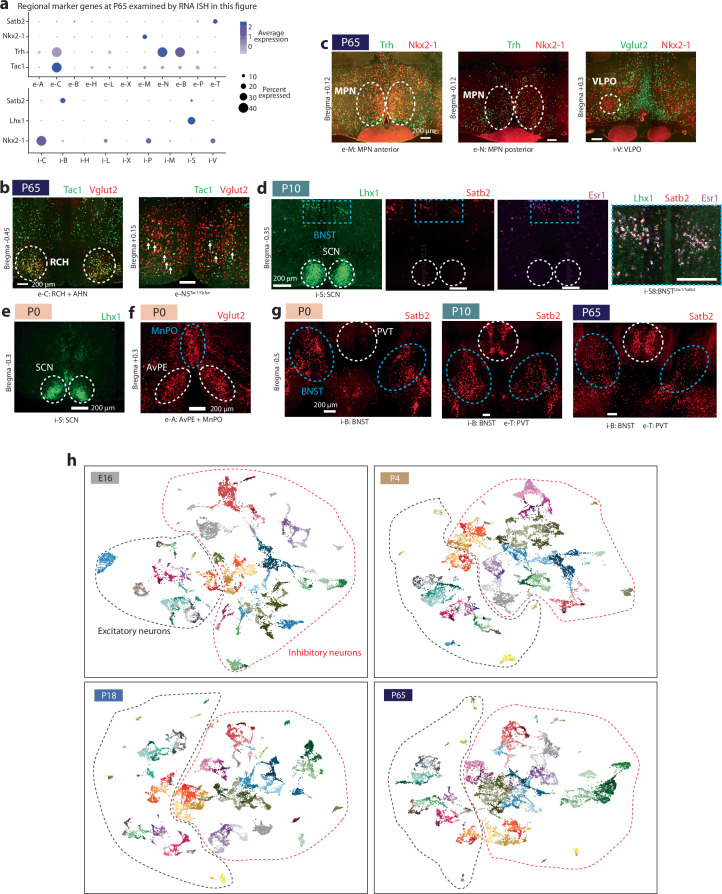
RNA in situ hybridization to confirm cell type localization and changes across age. (**a**) Regional markers at P65, for genes stained using ISH in subsequent panels of this figure. (**b**) Left: Tac1+ Vglut2+ neurons residing in RCH. Right: A small population of Tac1+ Vglut2+ neurons reside in MPN, corresponding to e-N5^Tac1/Qrfpr^ (arrows, double-positive neurons). (**c**) Trh and Nkx2-1 differentiate anterior and posterior MPN excitatory neurons. Left: Nkx2-1+ and Trh- cells reside in anterior MPN. Many of these neurons are Vglut2+ (data not shown, but co-stained). Middle: Trh+ and Nkx2-1- cells reside in posterior MPN. These neurons are also Vglut2+ (data not shown, but co-stained). Right: a population of Nkx2-1+ and Vglut2- neurons demarcates VLPO. (**d**) Left: Lhx1 is a marker for SCN neurons at P10 (and other ages). Other panels identify a population of BNST neurons, termed i-S8:BNST^Lhx1/Satb2^, that co-expresses Lhx1, Satb2, and Esr1. (**e**) Lhx1 is a marker for SCN neurons also at P0. (**f**) Vglut2 staining shows the inverted-Y structure of AvPe/MnPO at P0, as in adults (panel **c**, right) (**g**) Satb2 is a marker for BNST at P0, P10, and P65, but for PVT only from P10 onwards, not at P65. (**h**) UMAPs colored by cell type ID at four separate ages. Excitatory and inhibitory neurons are plotted together and encircled by dotted lines (black = excitatory; red = inhibitory). See key in Fig. 1c for cell type color labels.

Louvain clustering and gene marker analysis identified the main cell classes among snRNA-seq profiles (Extended Data Fig. 1a and Methods). Clustering of neuronal profiles from adult (P65) samples resulted in 83 GABAergic (γ-aminobutyric acid-producing) and 64 glutamatergic cell types. We used label transfer^21^ to map P65 cells onto our previously published scRNA-seq and MERFISH atlas of adult POA^1^, revealing a high degree of correspondence (roughly 70% of cells mapping to the top matched cluster on average) between our de novo clusters and previously identified cell types, including those with known roles in homeostasis or adult social behaviours (Fig. 1b, Extended Data Fig. 1b,c and Supplementary Table 1). In some cases (26 out of 63 reference scRNA-seq clusters), individual clusters from the reference atlas were split into several clusters in our data, indicating increased resolution. Supervised hierarchical clustering based on correlation and inferred spatial information from MERFISH label transfer revealed that cell types within a POA subregion share transcriptional signatures. We therefore defined a two-tier classification, with the first tier corresponding to subregional identity and the second tier corresponding to cell type (Fig. 1c and Supplementary Table 2). Cell type names denote (1) subregional identity (for example, ‘i-M’ for inhibitory MPN or ‘e-P’ for excitatory paraventricular hypothalamic nucleus (PVN)), (2) specific subregional localization if known (for example, i-M1:MPN/StHy (striohypothalamic nucleus)) and (3) marker genes based on both literature and this study (for example, i-M1:MPN/StHy^Gal/Calcr^). Some cell types did not map to the published POA atlas due to localization outside the MERFISH imaging area (Extended Data Fig. 2a–g).

**Extended Data Fig. 3:**
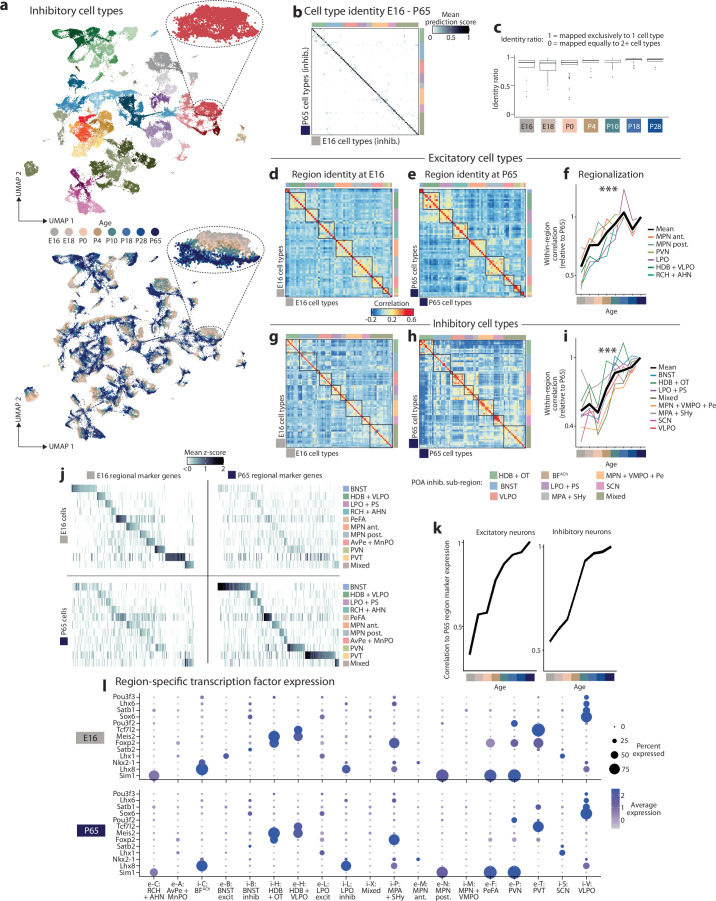
Region-specific gene expression changes across age. (**a**) UMAP across all 8 ages for inhibitory neurons. Upper, colored by cluster (key in Fig. 1c, upper); lower, colored by age. Inset: gradient across age for one cluster. (**b**) Mapping of E16 onto P65 clusters using canonical correlation analysis, for inhibitory neurons. Prediction score is averaged across cells within a cluster. Note that almost all E16 cell types map to a single P65 cell type, with very few off-diagonal elements. (**c**) Identity ratio quantifies how well clusters from young ages computationally map onto adult clusters: identity ratio of 1 denotes complete mapping to a single cluster, and identity ratio of 0 denotes equal mapping to 2 or more clusters. Each data point represents a single cluster, averaged across all cells. Data shown for excitatory cell types. Boxplots show median, interquartile range, and 1.5 * interquartile range, as well as all outliers beyond this range shown as points. (**d**) For excitatory neurons, correlation between pseudobulked gene expression of all clusters at E16. Colored bars on top and right indicate sub-region (excitatory sub-region key in Fig. 1d). Boxes demarcate cell types within the same POA sub-region. (**e**) As in (**d**) but at P65. Note that at P65, there are higher correlation values between clusters within the same sub-region (within the boxes, quantified in (**f**)) and lower correlation values between clusters in different sub-regions (between boxes). (**f**) For excitatory neurons, correlation between clusters within a sub-region (i.e. within the boxes in panels (**d-e**)), at each age, normalized to correlation at P65. ***p = 8.68 × 10^−5^ for one-way ANOVA, effect of age. (**g-i**) As in (**d-f**), but for inhibitory neurons. (**i**) p = 3.23 × 10^−13^, one-way ANOVA, effect of age. (**j**) Marker genes specific to excitatory neuron sub-regions, found at either E16 but not P65 (left) or P65 but not E16 (right), plotted for E16 cells (upper) or P65 cells (lower). (**k**) Using regional markers at P65 that are not markers at E16 (genes on right panel of (**j**)), correlation of regional expression at each age compared to P65. (**l**) Region-specific marker genes at E16 (upper) or P65 (lower).

Next, we asked how snRNA-seq profiles from developing POA map onto adult cell types. Using a similar label transfer approach^21^ (Methods), we sequentially mapped datasets from each developmental stage onto cluster identities at older stages (P28 to P65, then P18 to P28 + P65 and so on). This revealed one-to-one correspondence of nearly all cell types at younger ages to adult cell types, even at the earliest age E16 (Fig. 1d,e and Extended Data Figs. 2h and 3a–c), indicating that the near full complement of cell types is diversified before birth. Still, each cluster appeared in UMAP space as a temporal gradient of cells ordered across ages (Fig. 1e and Extended Data Fig. 3a), suggesting gradual maturational changes in gene expression on top of stable cell type identities.

**Extended Data Fig. 4:**
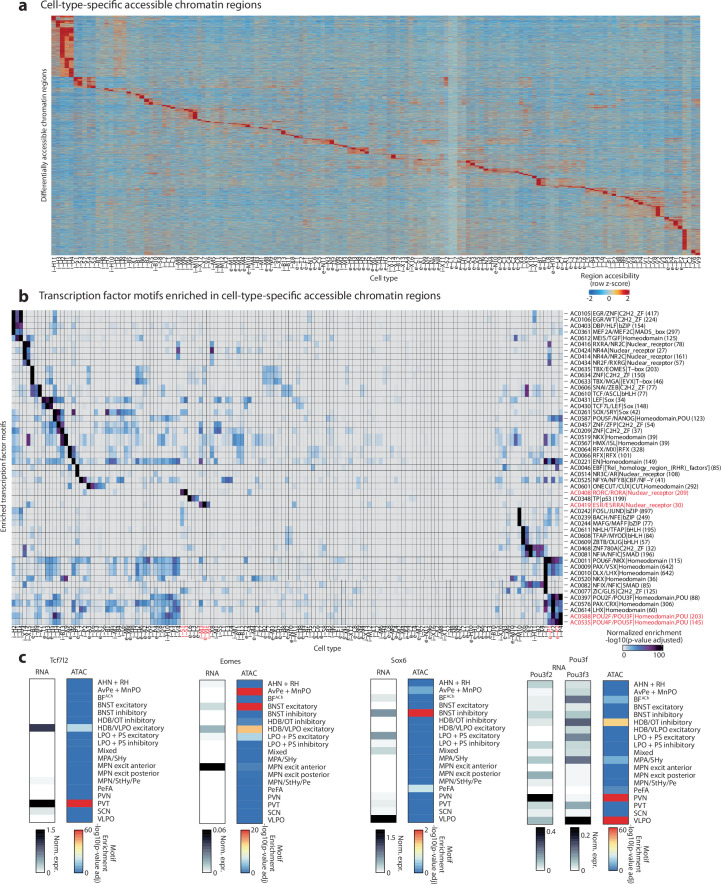
ATAC-seq identifies cell-type- and region-specific transcription factor motifs. (**a**) Differentially accessible regions (DARs) of chromatin specific to each cell type. (**b**) Cell-type-specific transcription factor motif enrichment, identified from cell-type-specific DARs. Motifs and cell types discussed in the main text are highlighted in red. Motif enrichment determined by hypergeometric test (Methods). (**c**) Examples of transcription factor motifs in accessible chromatin (ATAC-seq data) enriched amongst anatomical sub-regions that match with region-specific gene expression.

We next examined paired snATAC-seq libraries, using cell type identities from snRNA-seq. We determined cell type-specific genomic loci of chromatin accessibility and transcription factor motifs enriched in those loci (Extended Data Fig. 4a,b), identifying both known and novel candidate transcriptional regulators of cell identity. For example, the motif for the transcription factor family ROR/REV-ERB was highly enriched in two SCN cell types, i-S1:SCN^Avp/C1ql3^ and i-S3:SCN^Cck/C1ql3^, adding cell type specificity to a previously identified role for REV-ERB in SCN control of circadian insulin sensitivity in humans^22^. The oestrogen receptor *Esr1* motif was enriched in cell types with known sex differences in function or cell number^1,6^. This dataset thus represents a valuable atlas of candidate transcriptional regulators determining cell type identity.

We next assessed regional identity signatures during development. In the adult, clusters belonging to the same POA subregion were transcriptionally correlated (Extended Data Fig. 3e,h) whereas at E16, within-region correlations resembled those at P65, albeit less defined (compare boxed regions in Extended Data Fig. 3d,e,g,h). With age, these correlations strengthened, especially between P0 and P10 (Extended Data Fig. 3f,i). Subregion-specific marker gene expression also gradually resembled adult patterns, especially between P0 and P10 (Extended Data Fig. 3j,k). We confirmed these findings for specific genes and subregions using in situ hybridization (Extended Data Fig. 2a–g). Altogether, our data show that, between P0 and P10, the POA undergoes a regionalization process by which cell types within a given subregion adopt common transcriptional subregion-specific signatures, similar to the dynamic arealization process occurring in cortex^23^. Many regional transcription factors maintained regional specificity from E16 to P65 (Extended Data Fig. 3l) with some showing region-specific enrichment of motif accessibility in snATAC-seq data, thus potentially orchestrating regional identity (Extended Data Fig. 4c).

**Extended Data Fig. 5:**
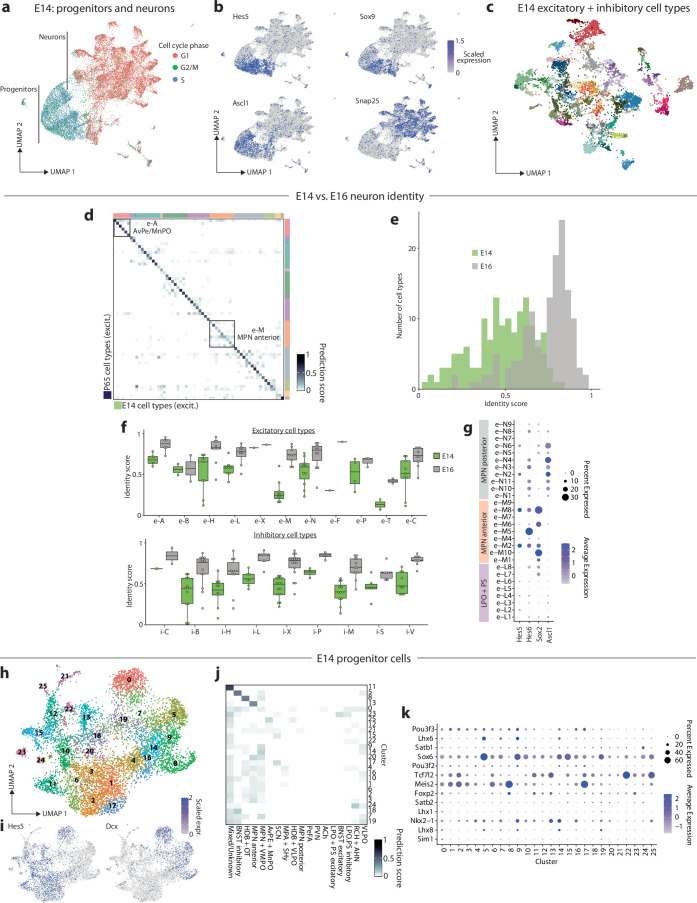
Analysis of E14 snRNA-seq data reveals differentiating neurons and progenitor cells. (**a**) UMAP of E14 cells colored according to assigned cell cycle phase, based on cell cycle marker expression. (**b**) Expression of progenitor cell markers Hes5, Sox9, and Ascl1, and mature neuronal marker Snap25. (**c**) UMAP of E14 excitatory and inhibitory neurons colored by mapped cell type (colors match legend in Fig. 1c). Compare to Extended Data Fig. 2h. (**d**) Mapping of E14 onto P65 clusters using canonical correlation analysis, for excitatory neurons. Prediction score is averaged across cells within a cluster. Note weaker on-diagonal elements and stronger off-diagonal elements compared to E16, shown in Fig. 1d. Boxed areas point out regions of interest: AvPe/MnPO cell types are fairly well differentiated at E14, similar to E16, whereas MPN anterior cell types are less differentiated at E14 than at E16. (**e**) Distributions of identity score, defined as the average prediction score given to the assigned cell type, for E14 and E16 cell types. E16 identity scores correspond to the values on the diagonals in Fig. 1d and Extended Data Fig. 3b. (**f**) Identity scores (as defined in legend for (**d**)) at E14 and E16, separated by POA sub-region. Certain sub-regions, such as excitatory MPN anterior neurons (e-M), appear less differentiated than others at E14 but not E16. Boxplots show median, interquartile range, and 1.5 * interquartile range calculated across cell types, with cells from n = 4 E14 and n = 2 E16 biological replicates. (**g**) Expression of progenitor cell markers in a subset of E14 cell types. MPN cell types express higher levels than LPO + PS cell types. (**h**) UMAP of E14 progenitor cells, colored according to unsupervised clustering results. (**i**) Expression of progenitor marker Hes5 and migrating neuron marker Dcx. (**j**) Mapping of E14 progenitor clusters onto E16 brain region labels using canonical correlation analysis. Prediction score is averaged across cells within a cluster. (**k**) Expression of region-specific marker genes defined at E16 and P65 (as in Extended Data Fig. 3l) among E14 progenitor cell clusters.

The early diversification of POA cell types prompted us to examine E14, a stage in which neurogenesis is still unfolding^20^ (Extended Data Fig. 5). In total, 24,316 nuclei were classified as either neurons or progenitors using known markers^24,25^ (Extended Data Fig. 5a,b). Among neurons, cells corresponding to specific adult populations could be identified but with less clear mapping than at E16, suggesting incomplete differentiation (Extended Data Fig. 5c–f). In particular, i-M1, i-M2 and i-M3, which are molecularly related but involved in distinct adult social behaviours, as well as anterior MPN excitatory neurons, were among the least differentiated (i-M and e-M classes; Extended Data Fig. 5f). At E14, MPN excitatory neurons also retained high expression of progenitor markers such as *Hes5*, *Hes6*, *Sox2* and *Ascl1*, which were lacking in other areas such as lateral POA (LPO) (Extended Data Fig. 5g), consistent with neurogenesis occurring later in medial compared to lateral subregions^20^. Progenitor cells split into 26 clusters and two main groups, one expressing high levels of progenitor markers such as Hes5 and the second expressing markers for cell migration such as *Dcx* (Extended Data Fig. 5h,i), perhaps representing newborn migrating cells. Progenitor clusters largely failed to map to any subregion, except outside POA such as the HDB, OT and BNST (Extended Data Fig. 5j). Finally, region-specific transcription factors at E16 through adulthood did not show cluster-specific expression in E14 progenitor cells (Extended Data Fig. 5k, compare to Extended Data Fig. 3l), and instead showed either widespread low or high expression, as has been described for early somatosensory neuron development^26^. Altogether, these data indicate that, in contrast to more posterior hypothalamic regions^25^, neuronal progenitors in POA lack mature regional distinctions, which they rapidly adopt after neurogenesis, together with cell type identities.

Next we identified, for each cluster, developmentally differentially expressed genes (devDEGs). Some devDEGs were shared across many clusters, indicative of common maturational changes, whereas others were specific to only one or a few clusters (Fig. 1f). Accordingly, we classified each devDEG as shared, cell type-specific or intermediate. This revealed roughly 2,500 devDEGs shared across nearly all cell types and up to 5,000 extra devDEGs with varying degrees of cell type specificity (Fig. 1f). To examine developmental timing, we fit trends to devDEG changes across age using a generalized additive model, pooled devDEG trends across cell types, and clustered them according to their dynamics^27^ (Fig. 1g). Some devDEG clusters represented genes that showed increasing or decreasing expression at specific ages, whereas others showed more complex bimodal patterns (Fig. 1h). Gene Ontology (GO) analysis revealed devDEG clusters enriched in processes relevant for neuronal development (Fig. 1i), indicating age-specific changes in functions such as axon guidance, cell adhesion, synapse assembly and synaptic signalling.

In summary, our dataset establishes a multiomic atlas of POA development comprising 147 neuronal populations distributed among 20 subregions, and identifies a dynamic regionalization process, candidate transcription factors regulating identity and cell type-specific gene expression changes pertinent to circuit maturation.

## POA cell types show diverse maturation timelines

**Fig. 2:**
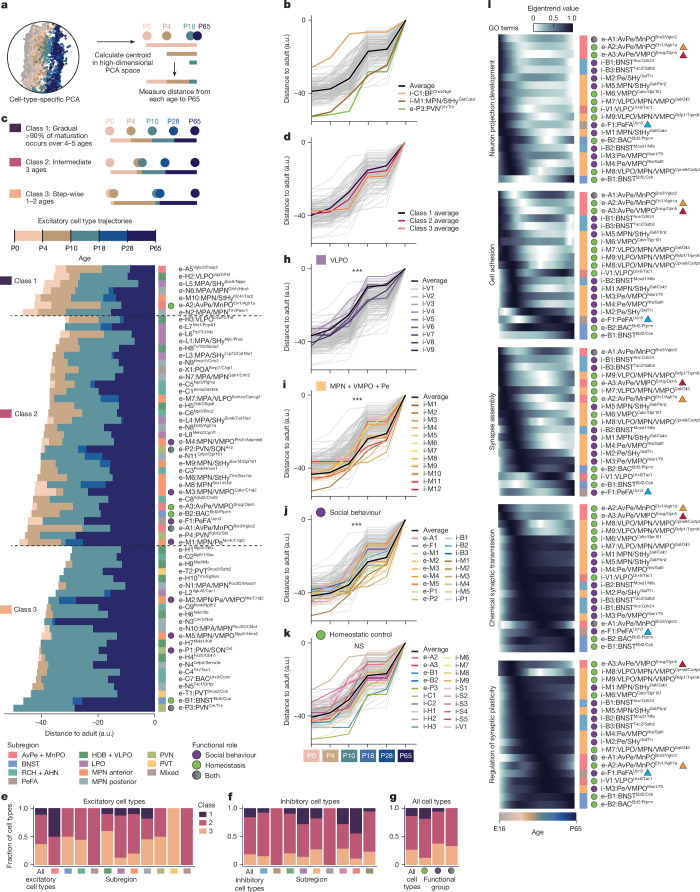
Developmental trajectories of POA cell types. **a**, Strategy for quantifying maturity as distance between each age’s centroid and the adult centroid in PCA space, calculated separately for each cell type. **b**, Distance trajectories across age for all cell types (grey), average (black) and extreme cell types of interest (coloured). a.u., arbitrary units. **c**, Top, schematic showing different trajectory classes. Bottom, distance trajectories across excitatory cell types. Trajectories were split into three classes depending on whether 90% of the maturation occurs across 4–5 ages (class 1, gradual), three ages (class 2, intermediate) or 1–2 ages (class 1, stepwise). **d**, Distance trajectories averaged across each class. **e**, Fractional class distribution of excitatory cell types, split by subregion. **f**, As in **e** but for inhibitory cell types. **g**, Fractional class distribution of all cell types or split by functional group. **h**,**i**, Distance trajectories for inhibitory VLPO (**h**) or MPN + VMPO + Pe (**i**) cell types. ***P < 0.0001 for two-way analysis of variance (ANOVA), effect of cell type subset. *P* = 2.15 × 10^−11^ (**h**) and 5.5 × 10^−15^ (**i**). **j**,**k**, Distance trajectories for cell types with roles in social behaviour (**j**) or homeostatic control (**k**). ***P < 0.0001 or NS (not significant) for two-way ANOVA, effect of cell type subset. *P* = 1.39 × 10^−14^ and 0.541. **l**, Eigentrend values summarize expression for devDEGs in select GO terms across age, for clusters of interest. Red, blue, and orange triangles indicate clusters referred to in the text as showing especially early (red, orange) or late (blue) changes.

**Extended Data Fig. 6:**
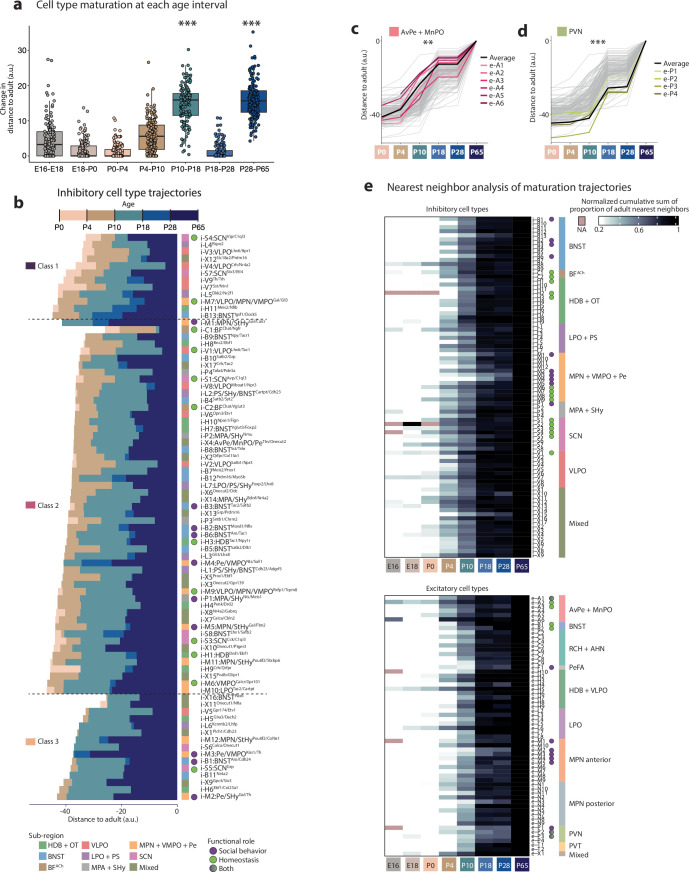
Maturational trajectories of POA cell types. (**a**) Difference between distance to adult at each subsequent pairs of ages. Each point represent a single cell type. ***p < 0.0001 for one-way ANOVA with post-hoc Tukey’s test comparing those P10-P18 and P28-P65 to all other ages (except each other). Boxplots show median, interquartile range, and 1.5 * interquartile range, with cells from n = 4 biological replicates per age (except E16, n = 2). (**b**) Distance trajectories of inhibitory clusters. (**c**) Distance trajectories for AvPe + MnPO clusters. **p = 0.000198 for two-way ANOVA, effect of cell type subset. (**d**) Distance trajectories for PVN clusters. ***p = 1.44×10^−11^ for two-way ANOVA, effect of cell type subset. (**e**) Heatmaps of scaled cumulative sum of proportion of nearest neighbors that are nuclei from adult (P65). Upper: inhibitory cell types. Lower: excitatory cell types.

Homeostatic control and social behaviours change along various postnatal timelines. For example, mice begin independent thermoregulation around P10 and feeding around P18, whereas mating emerges after P28 (puberty). To assess whether distinct POA cell types show specific maturation trajectories, we quantified, for each cell type, the distance in gene expression space^28^ between each pre-adult and adult age (Fig. 2a). This showed a wide diversity of maturational trajectories. For example, i-C1:BF^Chat/Ngfr^, a cholinergic basal forebrain cell type involved in arousal, is the most mature cell type at P0–P10, essentially already adult-like by P10 (Fig. 2b) (see Supplementary Table 1 for all citations for social and homeostatic functions). By contrast, e-P3:PVN^Crh/Trh^, which initiates the corticosterone stress hormone response, is relatively immature until after P28 (Fig. 2b). Furthermore, e-P3:PVN^Crh/Trh^ shows a stepwise trajectory, maturing at two key stages: P10–P18 and P28–P65. These two stages showed the most maturation across cell types (Extended Data Fig. 6a), suggesting weaning (roughly P16–P21) and puberty (roughly P28–P40) as key developmental events.

We examined these trajectories along two dimensions: mode of maturation (gradual versus stepwise; Fig. 2c–g and Extended Data Fig. 6b–e), and timing of maturation (early versus late; Fig. 2h–k), and asked whether trajectories along these dimensions correlated with features such as neurotransmitter, POA subregion or behavioural function. We classified each trajectory according to whether 90% of the maturational change occurred across 4–5 developmental stages (gradual, class 1); three stages (intermediate, class 2) or just 1–2 stages (stepwise, class 3) (Fig. 2c for excitatory and Extended Data Fig. 6b for inhibitory cell types). Nearly all cell types that matured in a stepwise manner (class 3) showed dominant P10–P18 and P28–P65 steps (Fig. 2c,d and Extended Data Fig. 6b). Glutamatergic cell types matured in a more stepwise fashion than GABAergic cell types (Fig. 2c,e,f and Extended Data Fig. 6b). Maturation class also varied by regional identity or functional role; for example, AvPe/MnPO cell types tended to mature gradually whereas cell types involved in social behaviour tended to have more stepwise trajectories (Fig. 2e–g). Some POA subregions showed strong biases towards early or late maturation. Cell types in VLPO and AvPe/MnPO, two subregions involved in homeostatic functions such as sleep, thirst and thermoregulation, were typically more mature than other cell types, at all ages (Fig. 2h and Extended Data Fig. 6c). By contrast, cell types in MPN/VMPO/Pe and PVN, subregions involved in social behaviour, were typically less mature than most other cell types, at all ages (Fig. 2i and Extended Data Fig. 6d). Across all subregions, cell types involved in adult social behaviours tended to be late maturing (Fig. 2j), whereas cell types involved in homeostatic control showed a wide range of maturation timing (Fig. 2k). Trajectory dependence on subregion and function was corroborated by an alternative measure of maturation, in which the proportion of adult nearest neighbours was quantified at each younger age^27^ (Extended Data Fig. 6e).

Focusing on devDEGs with ontology terms of interest, we identified an expected progression from neuronal projection development to cell adhesion and synapse assembly to synaptic function, as well as cell type-specific deviations from the norm (Fig. 2l). For example, consistent with the early maturation of AvPe/MnPO cell types (Extended Data Fig. 6c), the e-A2:AvPe/MnPO^Etv1/Agtr1a^ cell type involved in thirst and the e-A3:AvPe/MnPO^Sncg/Opn5^ cell type involved in thermoregulation showed the earliest peaks in several gene categories (Fig. 2l, red and orange triangles). By contrast, e-F1:PeFA^Ucn3^, which plays a key role in infanticide, and e-B1:BNST^Ebf2/Cck^, involved in anxiety and feeding, maintain expression of cell adhesion and synapse assembly genes unusually late (Fig. 2l, blue triangle). Overall, our data show diversity in cell type-specific maturational trajectories correlated with subregional identities and functional roles.

## Developmental changes in POA signalling networks

In adults, POA neurons affect behaviour through intra-hypothalamic and brain-wide signalling networks^3,4,6,19,29^. To examine when and how these signalling networks emerge during development, we quantified developmental gene expression changes in neuronal signalling systems, including neurotransmitters, monoamines, neuropeptides and hormones.

**Fig. 3:**
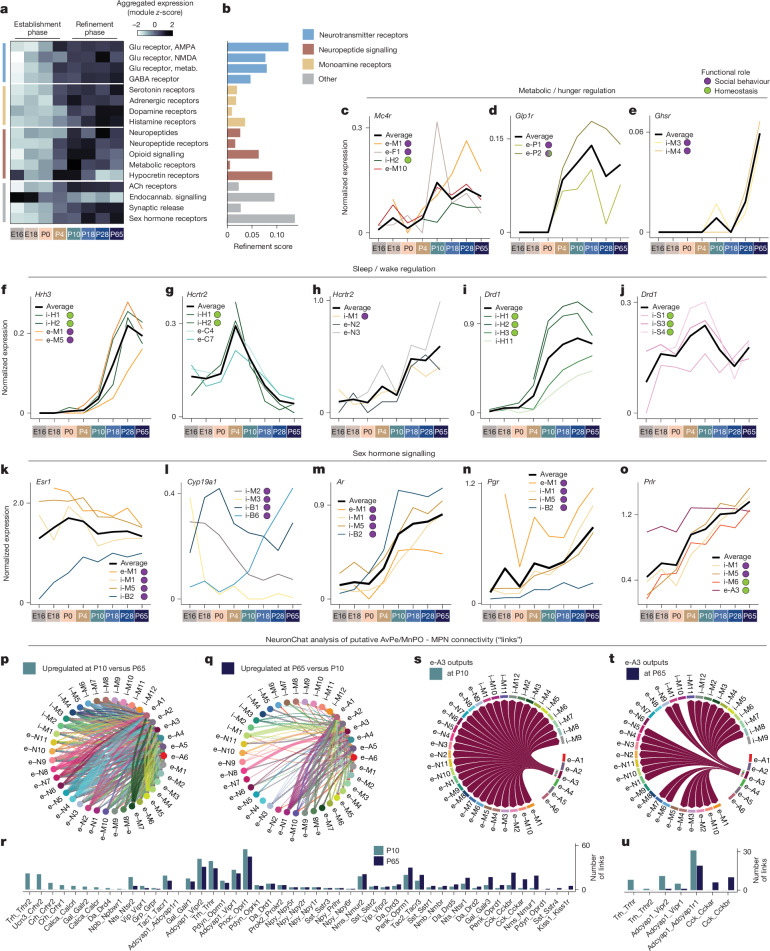
Maturational changes in POA neuronal signalling. **a**, Module score aggregating expression across various signalling gene categories, then *z*-scored across age. metab., metabotropic. **b**, Refinement score quantifies the number of genes in each category that show significant changes between P10 and P65 (refinement phase), normalized by the number of genes in each category. **c**–**e**, The log-normalized gene expression of receptors involved in metabolism and feeding: *Mc4r* (**c**), *Glp1r* (**d**) and ghrelin receptor *Ghsr* (**e**). Expression is averaged within each cluster across age, for example clusters (coloured lines). Black line shows average across example clusters. **f**–**j**, As in **c**–**e** for receptors involved in sleep–wake regulation: *Hrh3* (**f**), *Hcrtr2* with example cell types involved in sleep–wake (**g**) or social behaviour (**h**) and *Drd1* with example cell types in basal forebrain/HDB (**i**) or SCN (**j**). **k**–**o**, As in **c**–**e** for genes involved in sex hormone signalling: *Esr1* (**k**), *Cyp19a1* (encoding aromatase; **l**), *Ar* (**m**), *Pgr* (**n**) and *Prlr* (**o**). **p**,**q**, NeuronChat analysis of putative neuropeptide and monoamine connectivity between cell types in AvPe/MnPO and MPN at P10 versus P65. More links were upregulated at P10 (**p**) than at P65 (**q**). **r**, Number of NeuronChat neuropeptide and monoamine links identified at P10 or P65, sorted from left to right according to upregulation with age. **s**,**t**, Output links from e-A3 at P10 (**s**) or P65 (**t**). **u**, As in **r** but for e-A3 outgoing links only.

**Extended Data Fig. 7:**
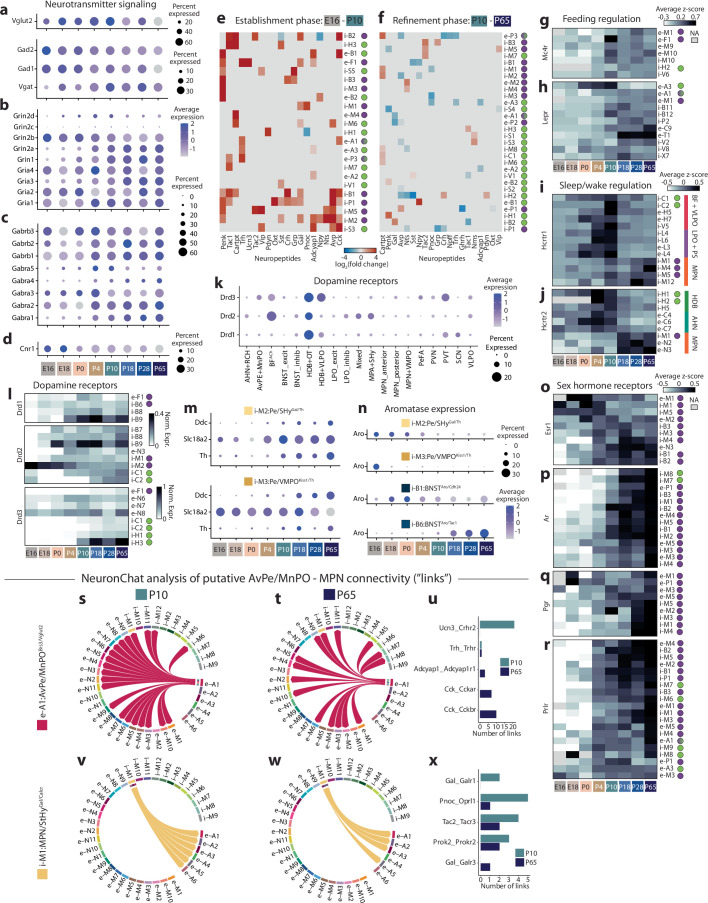
Developmental changes in neuronal signaling gene expression. (**a-d**) Dotplots showing expression across all neurons, separated by age, for glutamate and GABA synthesis and packaging enzymes (**a**), selected glutamate receptors (**b**), selected GABA receptors (**c**), or endocannabinoid receptor Cnr1 (**d**). (**e-f**) Heatmap showing significant increases (red) or decreases (blue) in expression of neuropeptide genes between E16 and P10 (**e**) or P10 and P65 (**f**). Gray indicates no statistical significance. (**g-j**) Heatmaps showing expression of selected genes involved in feeding behavior (g, Mc4r; h, Lepr) or sleep/wake behavior (**i**, Hcrtr1; **j**, Hcrtr2) across cell types in which those genes are enriched. Expression is z-scored within a cell type and then averaged across cells at each age. (**k**) Dotplot showing expression of dopamine receptors Drd1, Drd2, and Drd3 at P65, split by sub-region. (**l**) Heatmaps showing expression of Drd1, Drd2, and Drd3 across cell types in which these genes are enriched. Expression is log-normalized. (**m**) Dotplots showing expression of genes involved in dopamine synthesis and packaging in the two cell types that express all three of these genes, across age. (**n**) Dotplots showing change across age of aromatase expression in the four aromatase-enriched cell types. (**o-r**) Heatmaps showing expression of sex hormone receptors (**o**, Esr1; **p**, Ar; **q**, Pgr; **r**, Prlr) across cell types in which these genes are enriched and which have some functional annotations for social or homeostatic behaviors. (**s-x**) NeuronChat analysis of putative output connections from e-A1 (**s-u**) or i-M1 (**v-x**). Output cell types are shown at P10 (**s,v**) or P65 (**t,w**), with output links compared at these two ages in (**u,x**).

GABA and glutamate synthesis and vesicle packaging enzymes were highly expressed at E16 and showed little change with age (Extended Data Fig. 7a). Several GABA and glutamate receptor genes and genes for synaptic release machinery were expressed at E16 (Extended Data Fig. 7b,c). Neurotransmitters can serve non-synaptic roles during development^30^, a possibility we cannot rule out when interpreting gene expression data. The expression of GABA and glutamate receptor genes steeply increased from E16 to P4; after P4, overall levels (aggregated across genes and cell types) remained constant through adulthood (Fig. 3a). Some individual receptor genes, such as *Grin2a*, which encodes the GluN2A subunit of the NMDA (*N*-methyl-d-aspartate) receptor and is important for plasticity and neuronal maturation^31^, resembled changes to overall receptor levels, whereas others, such as *Grin2d*, which encodes the GluN2D subunit of the NMDAR, showed different dynamics, peaking at P4–P10 (Extended Data Fig. 7b). These results indicate two distinct phases of neurotransmitter receptor expression: (1) a global increase between E16 and P4, which we interpret as an establishment phase, and (2) reorganization of cell type- and receptor-specific gene expression between P10 and P65, viewed as a refinement phase.

**Extended Data Fig. 8:**
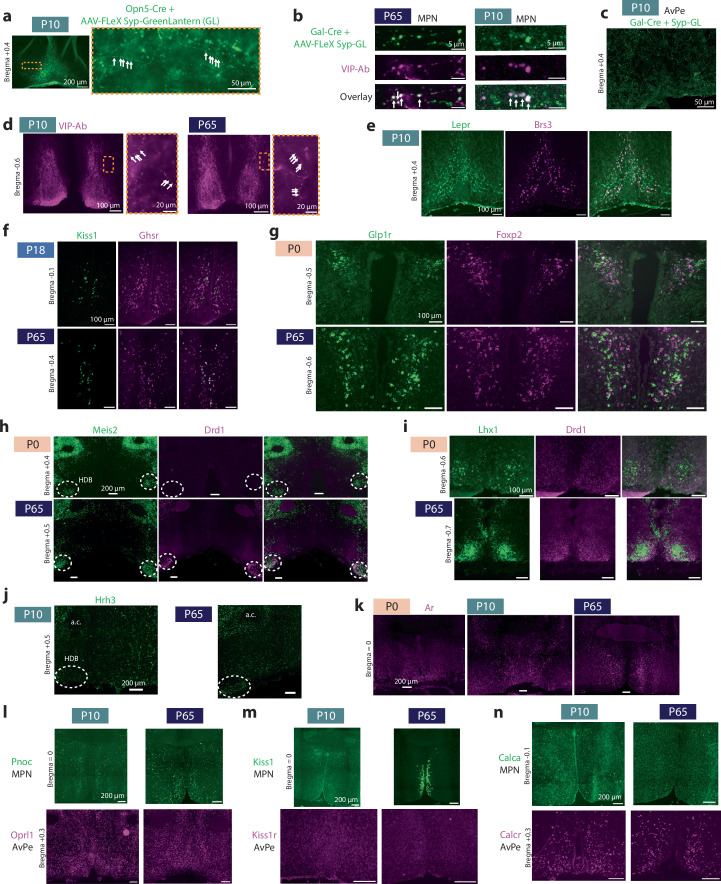
In situ validation of synaptic signaling gene expression changes. (**a**) mGreenLantern-tagged synaptophysin (Syp-GL) puncta (arrows) indicate putative presynapses in Opn5+ neurons in AvPe at P10. (**b**) Co-localization of synaptophysin and VIP protein puncta (arrows) in Gal+ neurons in MPN at P65 (left) and P10 (right). (**c**) MPN Gal+ neurons projecting to AvPe at P10 show dense synaptophysin puncta. (**d**) VIP puncta (arrows) at P10 (left) and P65 (right). (**e**) RNA in situ hybridization shows Lepr expression in Brs3+ and other AvPe neurons at P10. (**f**) Expression of Ghsr in Kiss1+ neurons at P65 (lower) but not P10 (upper). (**g**) Expression of Glp1r in Foxp2+ neurons of the PVN at P0 (upper) and P65 (lower). (**h**) Expression of Drd1 in Meis2+ HDB neurons at P65 (lower) but not P0 (upper). (**i**) Expression of Drd1 in Lhx1+ SCN neurons at P0 (upper) and P65 (lower). (**j**) Expression of Hrh3 in HDB neurons at P65 (right) but less so at P10 (left). (**k**) Expression of Ar is weak at P0, intermediate at P10, and widespread at P65. (**l**) At P10 and P65, Pnoc is expressed in MPN and Oprl1 is expressed in AvPe, consistent with potential Pnoc-Oprl1 signaling from MPN to AvPe as predicted by NeuronChat. (**m**) At P65 but not P10, Kiss1 is expressed in Pe, while Kiss1r is detectable at low levels in AvPe at both ages, consistent with P65 but not P10 Kiss1-Kiss1r signaling from MPN/Pe to AvPe as predicted by NeuronChat. (**n**) Calca expression is weak at P10 but nearly absent at P65 in MPN, while Calcr is expressed in AvPe at both ages, consistent with P10 but not P65 Calca-Calcr signaling from MPN to AvPe as predicted by NeuronChat.

Next, we examined changes in other signalling systems, including monoamines, neuropeptides and hormones. Nearly all gene sets showed patterns similar to neurotransmitter receptor genes, with an establishment phase during which expression increased from E16 to P4, followed by a plateau from P10 to P65 (Fig. 3a). Notable exceptions include genes encoding neuropeptides and dopamine receptors, which showed the most increased expression between P4 and P10, and histamine receptors, which increased in expression gradually until P18. Endocannabinoid signalling followed a different pattern altogether (Fig. 3a): the cannabinoid receptor gene *Cnr1* was already expressed at high, adult-like levels in most cell types at E16 (Extended Data Fig. 7d), suggesting that endocannabinoid signalling may be particularly important in developing POA as in other brain regions^32,33^. Between P10 and P65, most signalling systems showed constant overall expression, yet individual genes continued to change expression in particular cell types, consistent with a refinement phase (Extended Data Fig. 7e–r). To better assess these changes, we quantified a refinement score for each signalling system, a measure of how many significant expression changes were found between P10 and P65 (example shown for neuropeptides in Extended Data Fig. 7f). This revealed that monoamine and neuropeptide signalling showed low levels of refinement, compared to neurotransmitter receptors, sex hormone receptors or specific neuropeptide families such as opioid signalling or hypocretin receptors (Fig. 3b), consistent with protracted changes in functions such as sleep or social behaviour. To examine whether these gene expression patterns lead to mature synaptic proteins, we assessed the localization of synaptic vesicle marker synaptophysin (SYP), a commonly used marker of synapse development^34^. Using a viral strategy (Methods), we labelled SYP in Gal+ (MPN) or Opn5+ (e-A3) cell types and observed putative synaptic puncta in the MPN and AvPe at P10 (Extended Data Fig. 8a–c). We also immunostained P10 tissue for the neuropeptide VIP, chosen because of its expression in a single POA cell type, i-M5, and found that VIP puncta colocalized with SYP at P10 similarly to P65 (Extended Data Fig. 8b). Further, VIP puncta were visible in the SCN at P10 in a similar manner as at P65 (Extended Data Fig. 8d), consistent with snRNA-seq data (Extended Data Fig. 7e).

We next examined signalling systems with well-established roles in metabolism and hunger regulation, sleep–wake control and social behaviour, all of which show major postnatal changes. Receptors for signalling molecules affecting hunger regulation, such as melanocortins, ghrelin, leptin and Glp1, showed a staggered pattern of gene expression onset consistent with stepwise addition of regulatory mechanisms affecting feeding behaviour (Fig. 3c–e, Extended Data Fig. 7g,h and Supplementary Discussion). Receptors for signalling molecules affecting sleep–wake regulation showed distinct patterns (Fig. 3f–j, Extended Data Fig. 7i–m and Supplementary Discussion): the histamine receptor *Hrh3* showed a particularly late onset of expression at P18; hypocretin receptors showed a transient peak at intermediate ages in some cell types but not others and the dopamine receptor *Drd1* showed region-specific expression dynamics. Genes encoding receptors important for feeding and sleep–wake regulation were also expressed in cell types involved in adult social behaviours, in which they sometimes showed distinct expression dynamics (Fig. 3c–h). Finally, we found cell type- and age-specific onset of sex hormone receptor gene expression, and in the expression of aromatase (encoded by* Cyp19a1*) in four key cell types, suggesting that distinct cell types are involved in testosterone-to-oestrogen conversion at perinatal versus pubertal ages (Fig. 3k–o, Extended Data Fig. 7n–r and Supplementary Discussion). We validated many of these expression dynamics using RNA in situ hybridization (Extended Data Fig. 8e–k).

POA neurons are highly peptidergic, with individual cell types expressing a dozen or more neuropeptides. To understand the maturation of peptide signalling networks, we used NeuronChat^35^, a software package that infers neuronal connectivity based on statistically enriched peptide and receptor pair expression (for example, Vip-Vipr1), termed links. We focused this analysis on connectivity between MPN, a region largely but not exclusively involved in social behaviour, and AvPe/MnPO, which includes cell types associated with homeostatic functions such as thermoregulation and thirst. These two regions are known to project to one another^4,19,36^, with substantial connectivity already at P10 (refs. ^37,38^). To provide further evidence that MPN neurons may signal synaptically to AvPe/MnPO at P10, we virally expressed SYP in Gal+ MPN neurons and observed dense putative synaptic puncta in AvPe (Extended Data Fig. 8c). NeuronChat identified denser putative inter-connectivity at P10 than at P65 (Fig. 3p,q), with many neuropeptide links only found at P10 but not P65, such as Crh-Crhr1/2, Gal-Galr2 and Calca-Calcr (Fig. 3r). Fewer links were found at P65 but not P10, including Kiss1-Kiss1r associated with reproduction. We validated age-specific Calca-Calcr and Kiss1-Kiss1r expression, as well as persistent Pnoc-Oprl1 expression, using in situ hybridization (Extended Data Fig. 8l–n). These changes occurred across several cell types (Extended Data Fig. 7s–x); for example, e-A3:AvPe/MnPO^Sncg/Opn5^, an important player in thermoregulation, showed output links to all MPN cell types at P10, largely because of ubiquitous Adcyap1r1 expression (Fig. 3s–u). At P65, however, Adcyap1 signalling from e-A3:AvPe/MnPO^Sncg/Opn5^ was reduced and TRH signalling was lost; as a result, several MPN cell types no longer received putative input from e-A3:AvPe/MnPO^Sncg/Opn5^ (Fig. 3s–u). These changes may indicate pruning of exuberant connectivity between P10 and P65, as well as extensive neuropeptidergic signalling in developmental processes, as documented in other brain regions^39,40^. Further, cell types involved in social behaviour and homeostatic control show more neuropeptide inter-connections at P10 than at P65, consistent with a higher reliance on social interactions for homeostatic needs early in life^41,42^.

In summary, our analysis reveals complex expression dynamics in the development of POA signalling, such that distinct signalling networks emerge at different ages. Moreover, our data indicate substantial crosstalk between signalling systems and cell types involved in homeostasis and social behaviour.

## Development of sex differences in POA cell types

**Fig. 4:**
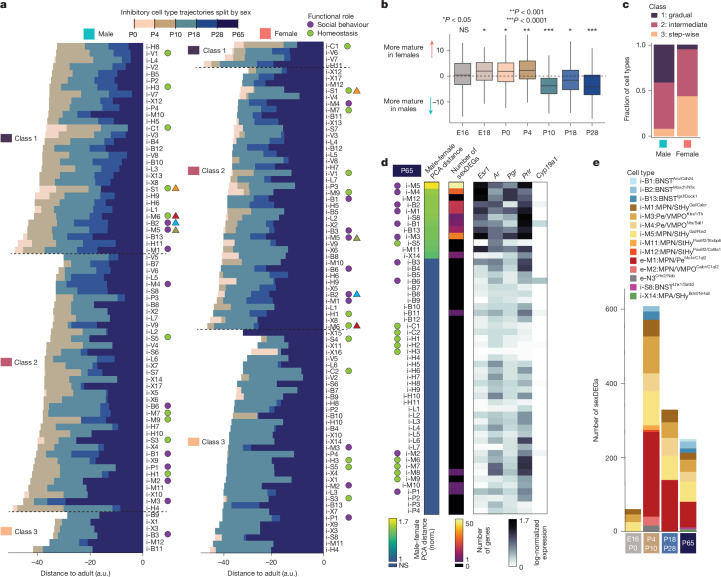
Sex differences in POA maturation. **a**, Distance trajectories among inhibitory clusters from male (left) or female (right) mice. **b**, Distribution of distance to adult at each age, subtracting male from female distance, per cluster. *P* value indicates results from two-sided *t*-test, male versus female distances, across all cell types. *n* for increasing age: 124, 135, 132, 141, 140, 143 and 141 cell types examined over two independent experiments per sex. Boxplots show median, interquartile range and 1.5× interquartile range. *P* values for increasing age: 0.399, 0.0038, 0.0022, 0.0001, 6.9 × 10^−8^, 0.035 and 5.63 × 10^−9^. **c**, Fractional class distribution of cluster distance trajectories. **d**, At P65, sex differences in inhibitory clusters measured by the distance between male and female sample centroids in PCA space (normalized (norm.) to intra-sex distance) (left) or the number of sexDEGs detected (middle). Right, averaged gene expression of sex hormone receptors. **e**, Number of sexDEGs detected at a given age for all clusters that show significant sex differences.

**Extended Data Fig. 9:**
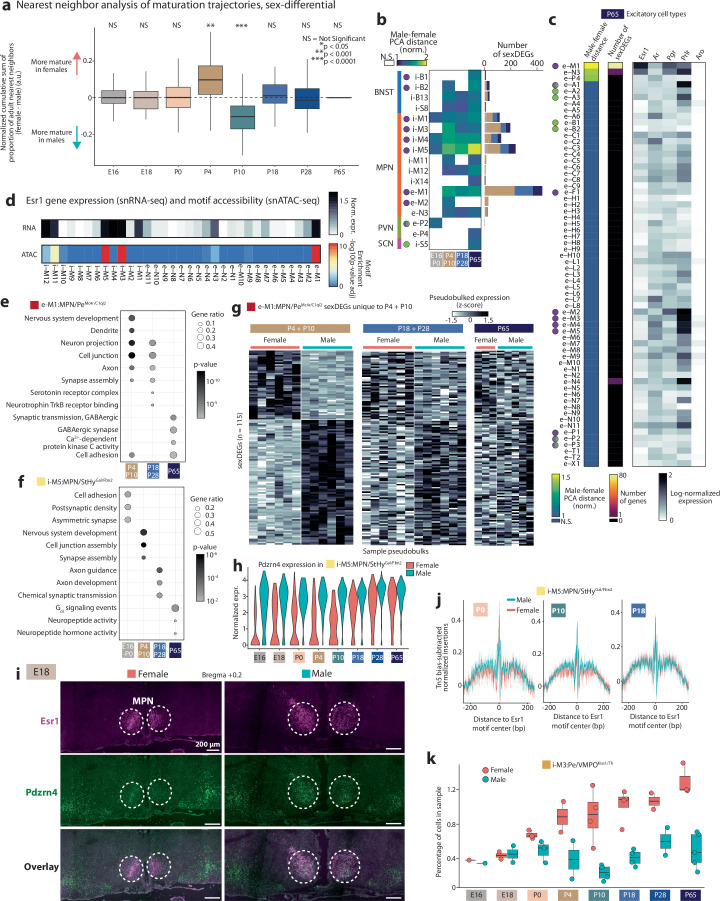
Age- and cell-type-specific sex differences in POA gene expression. (**a**) Difference between male and female proportion of adult nearest neighbors at each age. p-value indicates results from two-sided t-test, male versus female distances across all cell types. Boxplots show median, interquartile range, and 1.5 * interquartile range, with cells from n = 2 biological replicates per sex per age. p = 0.0002 (P4) and 3.64×10^−6^ (P10). (**b**) Left: All clusters that show significant sex differences in at least one age group according to male-female PCA distance. Right: number of sexDEGs among these clusters per age. N = 6-7 female and 6 male samples at each age, excepting P65 with 3 female and 5 male samples. N.S. = not significant. (**c**) At P65, sex differences in excitatory clusters measured by distance between male and female sample centroids in PCA space (normalized to intra-sex distance; left column) or number of sexDEGs detected (middle column). Grayscale matrix on the right indicates sex hormone receptor expression. N.S. = not significant. (**d**) Esr1 gene expression (upper) and cell-type-specific motif enrichment in accessible chromatin (lower) amongst MPN cell types. (**e-f**) Top GO terms for sexDEGs found only at specific ages in e-M1 (**e**) or i-M5 (**f**). (**g**) Gene expression values for 115 sexDEGs detected at P4-P10 but not other ages in e-M1, pseudobulked across cells within samples (each column represents a single sample). Control samples from mutant/control experiments included to increase sample number. (**h**) Violin plots showing expression of Pdzrn4, a sexDEG, in i-M5 neurons at all 8 ages separately in males and females. (**i**) RNA in situ hybridization confirms stronger expression of Pdzrn4 in males than in females in Esr1+ neurons at E18. (**j**) Bias-corrected ATAC-footprints centered around the Esr1 motif binding site, quantified separately for male and female i-M5:MPN/StHy^Gal/Fbn2^ cells at P0 (left), P10 (middle), or P18 (right). (**k**) Percentage of cells identified as i-M3:Pe/VMPO^Kiss1/Th^ in each sex at each age. Each point represents a single sample (3 POAs pooled). Boxplots show median, interquartile range, and 1.5 * interquartile range, with cells from n = 2-4 biological replicates per sex per age (except E16, 1 replicate per sex per age).

The POA and surrounding regions are hotspots of transcriptional sex differences in the brain^43^. We found widespread differences in maturation timing in males versus females (Fig. 4a–c and Extended Data Fig. 9a). POA cell types were more mature in females than males at E18–P4 but became more mature in males between P4 and P10–P28 (Fig. 4b and Extended Data Fig. 9a). Male-biased maturation at P4–P10 coincides with transcriptional changes resulting from the perinatal testosterone surge in males^44^. Cell types in females showed more pronounced maturation at P28–P65 (Fig. 4a), during puberty. Beyond maturation timing, sex also affected whether cell types matured gradually or stepwise. Owing to earlier and more pronounced maturation at P4–P10 and P18-P28 in males than in females, cell types in males showed more gradual maturation (class 1), whereas cell types in females matured largely in two steps (class 3), P10–P18 and P28–P65 (Fig. 4a,c). These sex differences were present across many cell types, including those with low or undetectable levels of sex hormone receptor expression. This may result from low sensitivity of snRNA-seq for lowly expressed genes, but also raises the possibility of indirect effects from sex hormone-responsive neurons on the maturation of other cell types.

Sex differences in gene expression are concentrated in the MPN, Pe and BNST, result from surges in circulating sex hormones at birth and puberty, and depend largely on the oestrogen receptor ESR1 (refs. ^43–46^). However, the relative contribution of birth and puberty remains unclear. Do some cell types show sex differences only following birth and others only on puberty? Do perinatal sex differences affect different neuronal functions from postpubertal ones? To address these questions, we first identified cell types showing sex differences in adults (P65), using two measures: (1) distance between male and female cells in high-dimensional principal components analysis (PCA) space^28^ and (2) the number of differentially expressed genes (DEGs) between males and females (sexDEGs)^44^. These measures converged on 15 cell types with significant sex differences in gene expression (Fig. 4d and Extended Data Fig. 9c). These cell types were enriched in sex hormone receptor expression, particularly *Esr1* (Fig. 4d and Extended Data Fig. 9c), showed* Esr1* motif enrichment in ATAC-seq data (Extended Data Fig. 9d) and, for many, were previously associated with sex differences in gene expression or function^1,44,45,47^ (Supplementary Table 1).

We next examined earlier ages. In total, 17 cell types showed sex differences at one or more ages (Fig. 4e and Extended Data Fig. 9b). Fourteen of these are located in MPN/Pe and BNST, and seven have previously been implicated in sex-differential social behaviours (Extended Data Fig. 9b). How does birth and puberty affect sex differences among these cell types? Altogether, these data show a notable increase in sex differences following birth (8 cell types and 547 sexDEGs gained), and a much smaller change in sex differences after puberty (4 cell types gained, which showed only weak sex differences, and 151 sexDEGs gained but 316 lost). Many genes showed sex differences in expression at P4–P10 but not later ages (Fig. 4e and Extended Data Fig. 9g).

We further examined sexDEGs in two key cell types that showed the strongest sex differences, e-M1:MPN/Pe^Mc4r/C1ql2^ and i-M5:MPN/StHy^Gal/Fbn2^, which were recently shown to have sex-specific roles in social behaviour^1,6^. Each cell type showed sexDEGs unique to specific ages, with sexDEGs at earlier ages related to neuronal development and sexDEGs at later ages related to neuromodulator signalling (Extended Data Fig. 9e,f). snATAC-seq transcription factor footprinting analysis revealed sex-specific chromatin accessibility of *Esr1* at P0 and P10 but not P18, which may partly explain dynamic changes in gene expression (Extended Data Fig. 9h–j). Finally, we found evidence for a perinatal emergence of sex differences in cell number for one cell type, i-M3:Pe/VMPO^Kiss1/Th^ (Extended Data Fig. 9k). These results are further described in the Supplementary Discussion.

In conclusion, our dataset shows widespread sex differences in the mode and timing of POA cell type maturation, with the largest effects seen in cell types known to be involved in social behaviour. These findings illustrate how sex hormones play a global yet cell type-specific role in shaping POA development.

## Sensory input affects POA cell type maturation

Proper maturation of brain circuits involved in sensory processing relies on spontaneous and sensory-evoked neuronal activity^7–10^. Whether sensory activity also affects the maturation of brain regions involved in homeostatic control or social behaviour, such as the POA, is unclear^17^. We assessed sensory modalities critical for POA function: somatosensation, including thermoregulation^4,36^, social touch^6,48^ and chemosensation, including the main olfactory system, essential for mating and aggression^49,50^, and the vomeronasal system, required for pheromone detection and the sex-specificity of social behaviours^18,51–53^. We also investigated dark-reared mice, in which the development of brain areas involved in vision is affected^7,10^, potentially affecting hypothalamic functions such as sleep–wake regulation and circadian rhythms. We performed single-nucleus sequencing on POAs from mice with defects in each of these sensory modalities, focusing on P10, P18 and P65, key stages for POA maturation (Fig. 2). For each mutant, we asked which cell types differed from controls using (1) distance between mutant and control cells in high-dimensional PCA space and (2) a random-forest classifier.

**Fig. 5:**
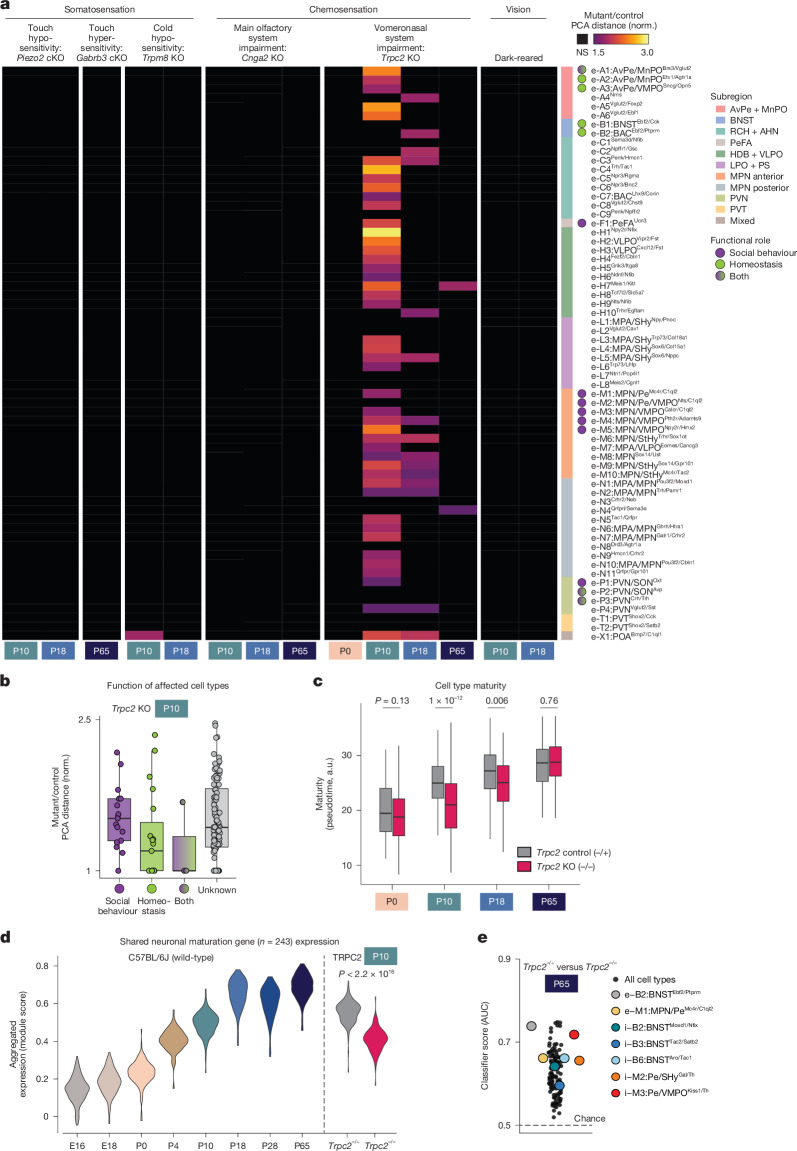
Sensory signals affecting POA maturation. **a**, Distance between mutant and control sample centroids in PCA space (normalized to intra-genotype distance) for excitatory clusters. Control samples included both C57BL/6J and control littermates. Sample numbers, left to right columns, mutant/control: 2/8, 4/10, 2/10, 2/8, 2/8, 2/8, 2/8, 2/7, 2/7, 2/8, 2/8, 2/7, 2/8 and 2/8. cKO, conditional knockout; KO, knockout. **b**, Mutant–control PCA distance for all clusters in TRPC2 P10 experiments, split according to functional role. **c**, Pseudotime mapping of *Trpc2* mutant (−/−) and control (−/+ littermates) across age. Higher pseudotime values correspond to higher maturity (more similar to adult cell types). Pseudotime is averaged within a cluster; boxplot shows distribution across all cluster averages. *P* value indicates results from two-sided *t*-test, mutant versus control cell types. *n* for increasing age: 82, 137, 131 and 128 cell types for controls, and 119, 135, 129 and 108 cell types for mutants, from *n* for increasing age of 2, 4, 2 and 2 independent experiments per genotype. Boxplots in **b** and **c** show median, interquartile range and 1.5× interquartile range. **d**, Aggregated expression of 243 core neuronal maturation genes in wild-type C57BL/6J cell types at each age (left) or *Trpc2*^−/+^ or *Trpc2*^−/−^ cell types at P10 (right). *P* value indicates results from two-sided *t*-test, mutant versus control cell types. **e**, Random-forest classifier approach (Augur) to identify cell types that show differences between *Trpc2*^−/+^ and *Trpc2*^−/−^ cell types at P65. Each point represents a single cell type. Cell types with roles in relevant social behaviours affected in *Trpc2*^−/−^ adults are shown with coloured dots. AUC, area under the curve.

**Extended Data Fig. 10:**
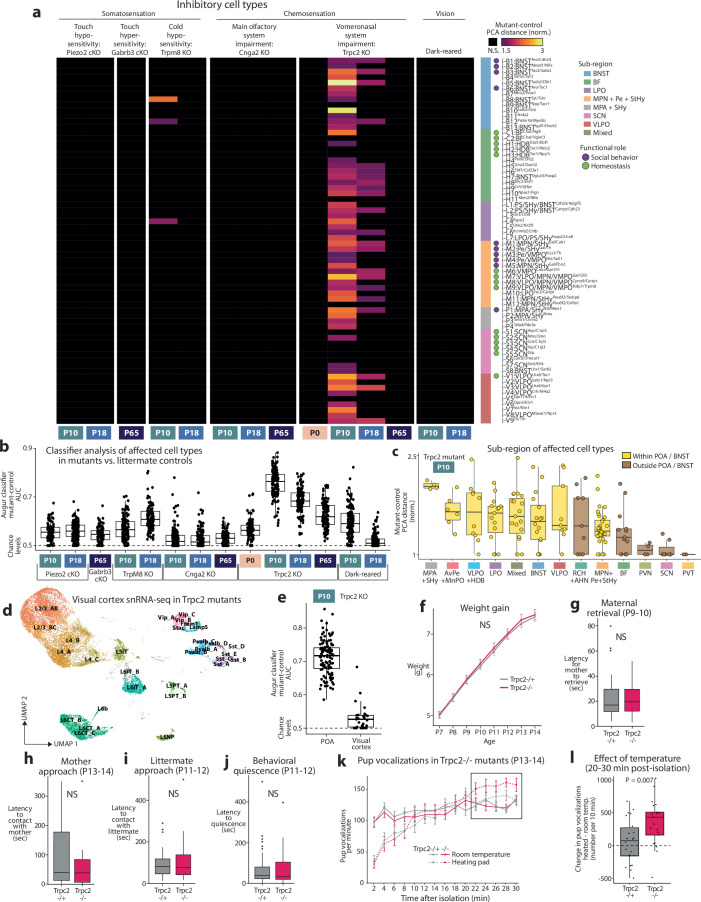
Effects of impaired sensory input on POA development. (**a**) Distance between mutant and control sample centroids in PCA space (normalized to intra-genotype distance) for inhibitory clusters. Control samples included both C57BL6/J and control littermates. Sample numbers, left to right columns, mutant/control: 2/8, 4/10, 2/10, 2/8, 2/8, 2/6, 2/7, 2/7, 2/8, 2/8, 2/7. (**b**) Random forest classifier approach (Augur) to identify cell types that show differences between mutant and control samples. Each point represents a single cell type. Number of biological replicates as described in (**a**). (**c**) Mutant-control PCA distance for all clusters in Trpc2 P10 experiments, split according to sub-region, and ordered from most to least affected. Data from n = 4 biological replicates per genotype. (**d**) UMAP showing identification of visual cortex neuronal subtypes in Trpc2 mutants and controls (pooled) at P10. (**e**) Random forest classifier (Augur) for replication of Trpc2 P10 POA (left) and visual cortex (right) from the same brains. Cells pooled from n = 2 biological replicates per genotype. (**f**) Weight of Trpc2−/− pups and Trpc2−/+ littermates at P7 to P14. n = 41 (Trpc2−/+) and 30 (Trpc2−/−). Error bars show standard error of the mean. (**g**) Latency for mothers to retrieve Trpc2−/+ or Trpc2−/− P9-10 pups when given a choice between the two. n = 48 (Trpc2−/+) and 48 (Trpc2−/−). (**h**) Latency for Trpc2−/+ or Trpc2−/− P13-14 pups to first contact their anesthetized mother. n = 26 (Trpc2−/+) and 22 (Trpc2−/−). (**i**) Latency for Trpc2−/+ or Trpc2−/− P11-12 pups to first contact with littermates. n = 36 (Trpc2−/+) and 20 (Trpc2−/−). (**j**) Latency for Trpc2−/+ or Trpc2−/− P11-12 pups to enter behavioral quiescence. n = 48 (Trpc2−/+) and 30 (Trpc2−/−). (**k**) Ultrasonic vocalizations emitted by isolated Trpc2−/+ or Trpc2−/− P13-14 pups, placed in a clean cage for 30 min at room temperature or with a heating pad underneath. Boxed portion quantified in (**l**). n = 22 (Trpc−/+), 21 (Trpc2−/−). Error bars show standard error of the mean. (**l**) Difference in the number of vocalizations between room temperature and heating pad conditions for each pup, at 20-30 min post-isolation (boxed portion in (**k**)). In all panels, boxplots show median, interquartile range, and 1.5 * interquartile range. For panels (**g-l**), p-value indicates results from two-sided t-test.

Mutants with conditional knockouts of *Piezo2* or *Gabrb3*, which lead to touch hypo- or hypersensitivity even before birth^54,55^, showed no POA cell types with significant differences compared to littermate controls (Fig. 5a for excitatory and Extended Data Fig. 10a for inhibitory cell types; both in Extended Data Fig. 10b). In* Trpm8* mutants strongly impaired in cold sensation^56^, four cell types showed significant differences at P10, including e-X1:POA^Bmp7/C1ql1^ that localizes to thermoregulatory nuclei AvPe/MnPO. In *Trpm8* mutants at P18, a more sensitive classifier approach showed differences in several cell types (Extended Data Fig. 10b), including several AvPe/MnPO clusters. Mice raised in complete darkness showed no POA cell types with significant differences at P10 or P18 (Fig. 5a and Extended Data Fig. 10a,b). Altogether, these data indicate minimal roles for somatosensory input, except potentially for cold sensation, and no role of visual inputs in POA transcriptomic maturation.

We next examined mutants with loss of main olfactory (*Cnga2* knockout)^57^ or vomeronasal (*Trpc2* knockout)^51^ function. *Cnga2* knockout mice showed no POA cell types with significant differences compared to littermate controls at P10, P18 or P65 (Fig. 5a and Extended Data Fig. 10a,b). By contrast, most cell types in *Trpc2* mutants showed a significant difference between mutants and controls at P10 (Fig. 5a and Extended Data Fig. 10a,b). These effects were absent at P0, largest at P10, smaller at P18, and small but detectable at P65 (Fig. 5a and Extended Data Fig. 10a,b), indicating a mostly transient effect that coincides with the timing of major maturational changes in POA (Figs. 1–4). Because *Trpc2* expression is restricted to the vomeronasal organ^58^, we ascribe this effect to impaired vomeronasal function.

Pheromonal information from vomeronasal sensory neurons reaches POA by means of accessory olfactory bulb projections to BNST and medial amygdala^59^. In *Trpc2* mutants at P10, cell types in BNST were particularly affected, as were cell types in various POA subregions, whereas cell types outside the POA in regions with little involvement in social behaviour, such as SCN or BF, were less affected (Fig. 5a and Extended Data Fig. 10a,c). Cell types involved in social behaviour were more affected than cell types involved in homeostatic control (Fig. 5b), consistent with the importance of vomeronasal signalling in social behaviour control and major vomeronasal input to the POA^59^. Some effects were also seen in cell types not expected to rely on vomeronasal signalling, for example VLPO^Tac1^ neurons involved in sleep and VMPO^Calcr^ neurons involved in fever generation and sickness behaviour, suggesting either unexpected vomeronasal connectivity or indirect developmental effects from neighbouring cell types. To validate these results and assess their specificity, we collected *Trpc2* mutant and control littermate brains at P10, dissecting both POA and visual cortex from the same brains. Most POA cell types were significantly different in *Trpc2* mutants versus control littermates, replicating our initial result, whereas visual cortex cell types were largely unaffected (Extended Data Fig. 10d,e).

POA cell types are most affected in *Trpc2* mutants at P10, and to a lesser extent P18 (Fig. 5a and Extended Data Fig. 10a,b). This could indicate improper developmental timing, such as transiently accelerated or delayed maturation, or an alternative developmental route that later converges towards the wild-type adult state. To address this, we quantified a pseudotime trajectory^60^ for each cell type using our wild-type dataset, which gives a maturation score to each cell. We then projected* Trpc2* mutant and littermate control cells onto the wild-type pseudotime axis to determine their maturation states (Fig. 5c). As expected, both *Trpc2* control and mutant cell types showed increasing pseudotime with age. However, at P10 and P18 but not P0 or P65, *Trpc2* mutant cell types showed significantly lower pseudotime values than controls (Fig. 5c), consistent with a transient developmental delay. Cell types in *Trpc2* mutants at P10 resembled those in littermate controls at P0 (Fig. 5c). Next, using wild-type data, we identified shared neuronal maturation genes (*n* = 243) that increased expression with age in all cell types (Fig. 5d, left). These genes showed significantly lower expression levels in P10 *Trpc2* mutants compared to littermate controls (Fig. 5d, right). We conclude that at P10, POA cell types in Trpc2 mutants lag behind in upregulating the expression of generic neuronal maturation genes, and that expression of these genes reaches normal levels by adulthood. In summary, most POA cell types, particularly although not exclusively those involved in social behaviour, require vomeronasal input for proper developmental timing.

Are there functional consequences to the transient developmental delay in POA transcriptional maturation at P10–P18? The involvement of POA in P10–P18 mouse behaviour is largely unstudied. First, we tested a variety of individual social (mother, littermate approach) and homeostatic (weight gain, sleep-like state) phenotypes in *Trpc2* mutant pups and found no significant effects (Extended Data Fig. 10f–j). Next, we tested two combined POA functions, temperature sensation and vocalization control. Mouse pups emit robust ultrasonic vocalizations when isolated from the nest (room temperature), but fewer vocalizations at thermoneutral (for example, heating pad) conditions^42^. *Trpc2* mutant pups showed increased vocalizations after 20–30 min isolated at warmer conditions (Extended Data Fig. 10k,l), consistent with affected POA circuits linking warm-sensing and vocalization control. In adults, a sensitive classifier analysis identified many cell types at P65 that were affected in *Trpc2* mutants compared to controls (Fig. 5e). Although affected cell types were not overall biased towards social or homeostatic function, they included several involved in adult social behaviours such as mating, inter-male aggression and infanticide (Fig. 5e), previously shown to be strongly impaired in adult *Trpc2* mutants^18,51,53^. Whether these more subtle differences in gene expression in POA cell types contribute to differences in *Trpc2* mutant adult behaviour should be examined in future work.

## Discussion

In this study, we explored transcriptional and chromatin dynamics underlying POA cell type development. POA cell types appear well-diversified at E16, soon after neurogenesis, consistent with a study in the Arcuate nucleus^61^ but different from developmental timelines in cortex^62,63^ or sensory systems^26,64^, where subtype diversification continues well beyond neurogenesis and into postnatal life. POA progenitors at E14 lacked mature regional identities, indicating that POA neurons gain regional and cell type identities shortly after neurogenesis. Postnatally, POA cell types undergo several key maturational steps. Between P0 and P10, subregional identity strengthens (Fig. 1), signalling networks are established (Fig. 3) and vomeronasal input ensures proper maturational timing (Fig. 5). Signalling networks are then reconfigured and cell types become increasingly adult-like, with two critical maturation stages identified as P10–P18 and P28–P65 (Fig. 2). P10–P18 just precedes weaning and has been identified in various brain regions as a key maturational time point molecularly and physiologically^65–67^. P28–P65 encompasses puberty, and cell types involved in social behaviour show extensive maturation at this age. Sex affects maturation at all time points, including the perinatal emergence of sex differences in Esr1^+^ cell types, widespread differential maturation trajectories and dynamic cell type-specific sex differences in gene expression on puberty (Fig. 4). Overall, our results indicate that the precise timeline of maturation is highly cell type-specific, occurring at different time windows and in a gradual or stepwise manner according to cell subregional identity, adult behavioural function and sex.

We examined POA cell type maturation in mutants affecting various sensory modalities and found little effect of somatosensory, visual or main olfactory impairment. Clear caveats are the lack of snRNA-seq sensitivity to lowly expressed genes, poor representation of some cell types, inclusion of few postnatal time points and partial removal of sensory inputs (for example, remaining touch signals above the neck in *Piezo2* conditional knockout mice or heat sensation in *Trpm8* knockouts). By contrast, we found a major effect on maturational timing for most cell types in animals with loss of vomeronasal function. This suggests that input-dependent plasticity during development occurs beyond sensory and cognitive systems, in brain regions classically assumed to be genetically hard-wired^17,68,69^. How POA maturation depends on vomeronasal input is a significant question for future work. In *Trpc2* mutants, vomeronasal neurons fail to respond to pheromones^51^, but show normal spontaneous neuronal activity, a key neurodevelopmental factor in other sensory systems. Whether late embryos and newborns sense vomeronasal cues has not been explored, nor is it known whether vomeronasal signals may affect POA neuronal activity in early life. Vomeronasal inputs reach some POA cell types and not others (for example, Gal+ but not Gnrh+ neurons^19,49^). The broad effects of *Trpc2* mutation on many POA cell types suggest that cell types receiving direct vomeronasal input may be initially affected and fail to release key signalling molecules, thus indirectly affecting the maturation of neighbouring cell types. NeuronChat analysis identified several POA clusters that signal through distinct neuropeptides at P10 versus P65 and may thus have key maturational roles. P0–P10 may be a critical time point in VNO-POA pathway development, and corresponds to the emergence of projections from BNST to POA^37,38^. In adults, we see small differences between *Trpc2* mutant and control POA cell types, with unclear relevance for adult function. Even a transient transcriptional difference during development can lead to permanent and functionally relevant differences in connectivity and other neuronal features^70^. We propose that ~P10 may represent a critical period for POA circuit development and sensitivity to vomeronasal input.

Altogether, our work shows that sensory input, sex, subregion localization and function impinge on POA cell type development. This points to a developmental process that is sensitive to external influences, such as vomeronasal input, sex hormone secretion and subregion-specific signalling. Furthermore, adult homeostatic or social behaviour function may determine developmental trajectories through genetically preprogrammed (for example, lineage-based) as well as environmental influences, such as vomeronasal and sex hormone inputs or effects associated with age-dependent life changes such as the onset of thermogenesis and independent feeding. The sensitivity of POA development to external inputs suggests underlying mechanisms by which early life experience may lead to long-lasting effects on social behaviour or homeostatic control. Thus, as proposed by Nikolaas Tinbergen in his ‘four questions’^13^, studies of development may help to address long-standing questions on the origin of instinctive behaviours.

## Methods

### Animals

Mice were maintained on a 12 h–12 h dark–light cycle with access to food and water ad libitum. The temperature was maintained at 22 °C and the humidity was controlled at 30–70%. All experiments were performed in accordance with National Institutes of Health (NIH) guidelines and approved by the Harvard University Institutional Animal Care and Use Committee. Except for experiments on mutant strains affecting sensory input, C57BL/6J mice (RRID:IMSR_JAX:000664) were used for all sequencing experiments. Mothers of all C57BL/6J experiments subjects were placed in a fresh cage when embryos were ~E12. For samples collected at P28 and P65, mice were separated from parents and opposite-sex siblings at P21 and group-housed, then kept separated from the opposite sex until dissection. C57BL/6J brains were collected during the first 3 h of the dark cycle. *Cnga2*, *Trpc2* and *Trpm8* mutants were treated the same, whereas *Piezo2* and *Gabrb3* conditional knockout mice and littermate controls were raised in another facility (Harvard Medical School) and thus brains were collected over a wider time window (2–7 h into the start of the light phase of 12 h–12 h light cycle). For dark-rearing experiments, pregnant mice were ordered from Charles River Laboratories (RRID:SCR_003792) and delivered to Boston Children’s Hospital’s Animal Behaviour and Physiology Core, where they were then housed either in constant darkness (cage changes performed with night-vision goggles) or standard 12 h–12 h light–dark conditions (control group). Biological replicates never contained brains from sibling pairs, although sibling pairs were occasionally pooled into one sample. All mutant experiments were performed with equal numbers of male and female samples, except for* Cnga2* knockouts in which only males were used due to the location of *Cnga2* on the X chromosome and impairments of *Cnga2* mutants in suckling and mating. *Cnga2* knockout mice, always males, were generated by crossing heterozygous mothers to FVB fathers leading to hemizygous male progeny. *Piezo2* conditional knockout mice were generated using *Cdx2-cre*, which drives recombination in neurons (including touch sensory neurons) and some peripheral tissues below cervical level C2, and either *Piezo2*^*fl*^/*Piezo2*^*fl*^ or *Piezo2*^*fl*^/*Piezo2*^−^. Brains from *Piezo2* conditional knockout mice were collected at P10, P18 or P19, and show motor defects, thus were frequently provisioned with extra food and gel packs placed on the cage floor; because of these defects, we did not raise animals beyond P19 for experiments in adults. We also did not profile *Trpm8* as adults, because thermoregulation maturation occurs mostly before P18. *Gabrb3* conditional knockout mice were generated using *Avil*-*cre*, which drives recombination in all dorsal root ganglion and trigeminal sensory neurons, and heterozygous *Gabrb3*^*fl/+*^ mice, which is sufficient to cause touch hypersensitivity^55^. Brains from *Gabrb3* conditional knockout mice were collected at P60–P70. Mutant and transgenic strains used in this study were previously published and include (with Jackson Laboratories (RRID:SCR_004633) numbers where applicable): *Trpc2* knockout mice^51^ (021208; RRID:IMSR_JAX:021208); *Cnga2* knockout mice^57^; *Trpm8* knockout mice^71^ (008198; RRID :IMSR_JAX:008198); *Piezo2*^*fl*^ (ref. ^72^) (027720; RRID:IMSR_JAX:027720); *Piezo2*^−^ (ref. ^73^); *Cdx2*-*cre*^74^) (009350; RRID:IMSR_JAX:009350); *Avil-cre*^75^, *Gabrb3*^*fl*^ (ref. ^76^), and *Opn5-cre*^77^. Sample sizes were chosen to match or exceed those in similar experiments from relevant studies.

### Single-nuclei sequencing

Brains were dissected (P10+) or whole heads were removed (E14 to P4), frozen in optimal cutting temperature medium and placed at −80 °C. The POA and surrounding regions were dissected on a cryostat at −15 °C using the Paxinos Developing and Adult Brain Atlases^78,79^ to determine locations for dissection based on landmarks. At all ages, tissue was collected from the anterior-most portion of MnPO to the posterior-most portion of the POA. To achieve this, after slicing away anterior material, thin (10–20 µm) sections were collected and examined under a dissecting microscope to identify landmarks. At E16 to P65, the anterior commissure on each brain hemisphere is distinctly visible and moves medially as the brain is sliced from anterior to posterior. When the medial edge of the anterior commissure lined up underneath the ventral tip of the lateral ventricle, we began collecting tissue for sequencing. At E14, the anterior commissure is not clearly visible; we instead began collecting tissue when we first saw the ventral tips of lateral ventricles reach their full extents. To collect tissue, we first used a razor blade to remove large blocks of tissue lateral to the lateral ventricles, and then sliced 200-µm-thick sections to collect roughly the ventral-most one-third of the brain into a 2-ml tube. At E14, we collected 800 µm; at E16–P4, we collected 1.2 mm and at P10–P65, we collected 1.4 mm. Dissected tissue was placed in a 2-ml tube and kept at −80 °C until the day of library preparation. For library preparation, 4–8 samples were prepared simultaneously, pairing mutant and control and male and female samples on the same day. Dissected tissues from three mice (C57BL/6J samples; two replicates per age per sex) or 2–3 mice (mutant and control samples; replicate numbers indicated within Fig. 5 legend) were pooled for each sample. Two female and two male samples were collected at each of the eight ages, except for E16 where only one female and one male sample were collected. Nuclei preparation was modified from 10X Genomics demonstrated protocol CG000375. Samples were dounce homogenized in a 3-ml Potter-Elvehjem Tissue Grinder in a 4 °C cold room in 0.1% NP40 lysis buffer, passed through a 70-µm filter (MACS Smart-strainer) and centrifuged at 500*g* for 5 min. All buffers contained 1 U µl^−1^ RNase Inhibitor (Sigma Protector) and centrifugation steps in nuclei preparation was done at 4 °C. Nuclei were resuspended in PBS with 2% BSA, then passed through a 20-µm filter (MACS Smart-strainer). 7-AAD was added and nuclei were separated from debris by fluorescence activated cell sorting. Following centrifugation, nuclei were permeabilized in 1× lysis buffer (10X Genomics, CG000375), then centrifuged and resuspended to a concentration of roughly 6,000 nuclei per µl, using a Luna Cell Counter. Downstream preparation of sequencing libraries was carried out using the 10X Genomics Multiome Kit. Libraries were sequenced on an Illumina NovaSeq 6000 S4 200 flowcell (eight samples per flowcell of either RNA-seq only or ATAC-seq only, targeting 200,000 reads per cell) using instructions provided by 10X Genomics Multiome protocol (read 1 = 26 bp, i1 = 10 bp, i2 = 10 bp, read 2 = 90 bp; ATAC read 1 = 50 bp, i1 = 8 bp, i2 = 24 bp, read 2 = 49 bp), to a saturation of at least 70% for each run. Paired-end sequencing with read lengths of 100 nt was performed for all samples.

### snRNA-seq analysis

Illumina sequencing reads were aligned to the mouse genome using the 10X Genomics CellRanger ARC pipeline with default parameters. For the C57BL/6J dataset, the mean numbers of unique molecular identifiers (UMIs) and genes per cell were 5,276 UMIs and 2,264 genes for all neurons. Raw reads and output are available on GEO at accession: GSE280964 (RRID:SCR_005012).

For initial analysis, we relied on the R package Seurat (RRID:SCR_016341) and standard data analysis practices. We filtered out nuclei with more than 400 or fewer than 100,000 UMIs, with fewer than 250 genes and with more than 20% of UMIs belonging to one gene. Although rare, nuclei with more than 10% mitochondrial or ribosomal UMIs and more than 1% IEG, apoptotic or red blood cell UMIs were also filtered out. We defined the main cell classes (glia, neurons) and separated out inhibitory and excitatory neurons using Seurat clustering and known marker gene enrichment.

#### Defining cell types

Cell types were defined separately among inhibitory and excitatory neurons using the same procedure. First, SCTransform^80^ was used to normalize all C57BL/6J data across all ages, regressing out percentages of mitochondrial UMIs, ribosomal UMIs and largest gene. Each cell from this dataset was then mapped to the published POA reference atlas^1^, separately for scRNA-seq and MERFISH reference atlases, using canonical correlation analysis-based label transfer^21^. Both reference datasets were normalized using SCTransform, and FindTransferAnchors was run using the SCTransform assays, PCA as the reference reduction and 50 principal components. TransferData generated predictions to reference atlas cell types using 50 principal components. Each cell was then assigned (1) a scRNA-seq, and (2) a MERFISH reference atlas predicted cell type, if the top prediction score exceeded 0.6; otherwise, a predicted cell type label was not assigned. These predicted cell type labels were used to guide subsequent clustering and cell type definition.

Then, P65 neurons were subset out to initially define cell types in adult neurons. Seurat functions RunPCA and FindNeighbors were run (150 principal components) and FindClusters (resolution 12) was used for an initial round of clustering. This generated clusters that often, but not always, had clear matches to POA reference atlas clusters, in terms of (1) majorities of cells predicted as a single MERFISH and/or scRNA-seq cell type (as described above) (Extended Data Fig. 1b), with scRNA-seq and MERFISH cell types matching as described in the POA atlas paper^1^; and (2) marker genes matching those described in the POA atlas paper^1^. Each cluster was assigned a cell type ID, with occasional grouping of 2–4 clusters that mapped to identical sets of reference atlas cell types. Grouping of clusters into subregional groups was determined by correlation analysis (Extended Data Fig. 3e,h) and previous knowledge about spatial location from MERFISH (Fig. 5c and supplementary figure 18 of ref. ^1^). In cases where cell types lacked clear matches to MERFISH data, we estimated location based on three-colour RNA scope staining and Allen Brain In Situ Hybridization Atlas data. Dendrogram plots were generated using the R package dendextend.

Having defined cell types at P65, we next mapped younger ages iteratively onto older ones (P28 to P65, then P18 to P28 + P65 and so on). For this, we subset each younger age, processed those data as described above for P65, then used FindTransferAnchors and TransferData as described above but with 150 principal components and with older ages as the reference atlas, to generate prediction scores for each cell. Cells with top prediction scores exceeding 0.8 were assigned to that cell type ID. Cells with top prediction scores exceeding 0.5 were also assigned to that cell type ID, as long as the prediction score was at least twice as high as the second-best prediction score. We chose these thresholds by exploring how well de novo clustering at each younger age generated clusters with homogenous predicted cell type ID labels. This procedure generated cell type IDs for 90% or more of cells at older ages, with lower percentages around P0 and earlier, which could be due to less precise dissection of smaller brains and thus inclusion of cells from brain areas not included at older ages. Prediction scores for cells that passed these thresholds are plotted in Fig. 1d and Extended Data Fig. 3b,c, averaged within a cell type. Identity ratio in Extended Data Fig. 3c is calculated using matrices in Fig. 1d and Extended Data Fig. 3b: the value on the diagonal (best cell type match) minus the top off-diagonal value (second-best cell type match), divided by the value on the diagonal. Cell types in mutant and control samples were determined using the same procedure, except that cells were label transferred onto all ages. This procedure assigned cell type IDs to 80–90% of cells in these samples, an accuracy similar to across-age mapping in C57BL/6J samples. The remaining 10–20% of cells not assigned IDs in this manner were removed from further analysis.

To identify cell types in visual cortex at P10 from *Trpc2* mutants and control littermates, we took a similar approach, using P8 and P14 visual cortex datasets as a reference atlas from ref. ^7^. We normalized P8 and P14 visual cortex data using SCTransform, used 150 principal components to perform label transfer and used cut-offs as described above to assign predicted cell type IDs. In our samples, 93% of cells were assigned predicted cell type IDs by this procedure.

#### Regionalization quantifications

Matrices in Extended Data Fig. 3d,e,g,h were calculated by averaging scaled RNA values among all cells within a cell type, then calculating the Pearson correlation between all such cell type vectors. Correlations among cell types within a region were averaged to generate Extended Data Fig. 3f,i. Seurat’s FindAllMarkers function was used to identify regional marker genes in Extended Data Fig. 3j–l, using an adjusted *P* value cut-off of 0.05. Plots were generated using ComplexHeatmap (RRID:SCR_01727) and pheatmap (RRID:SCR_016418) R packages.

#### devDEG analysis

The devDEG analysis presented in Fig. 1f–i (and used to calculate heatmap data in Extended Data Fig. 7e,f and refinement score in Fig. 3b) comprises a combination of R and Python scripts described previously^27^ (https://zenodo.org/records/7113422) that we adapted. Each cell type was subset across all ages, then sample-pseudobulked (*n* = 4 samples per age, except *n* = 2 at E16) and passed to the limma-voom pipeline^81^ from the edgeR (RRID:SCR_012802) package^82^ for differential expression analysis testing. To implement this, a Python v.3.10.9 conda environment was created to install and isolate all necessary packages. We subset Seurat objects by cell types (inhibitory, excitatory), then converted them first to H5 format (with SaveH5Seurat) and then to h5ad format for import in scanpy (with the Convert function from the SeuratDisk package https://github.com/mojaveazure/seurat-disk). We created cell type–data point batches using 02.pseudobulk-by-batch_limma-voom_data-prep.ipynb notebook. We detected DEGs with edgeR/limma-voom using 10_major_trajectory_devDEG.R R-script, defined as those with false discovery rate (FDR) < 5%. We fit linearGAM trends (generalized additive models) with LinearGAM (https://pygam.readthedocs.io, 11__dev-DEGs_age-trend-fits_rate-of-change.ipynb) for every gene across time points. We plotted trends using matplotlib (12__major-trajectory_dev-DEGs_stage-trend-fits.ipynb) (RRID:SCR_008624). We performed hierarchical clustering of DEG trends with 14__stage-trend-fits_clustering.ipynb and plotted the figures with matplotlib. This step was memory intensive and required up to 210 Gb of RAM for the largest dataset in our analysis. For each DEG cluster we performed Gene Enrichment Analysis (15__Trajectory-devDEG_Gos-terms.ipynb) and gprofiler2. We plotted cell type and time point expression heatmaps for genes in GO categories (16__Eigentrends-of-selected-GO-terms.ipynb).

For Fig. 2l, we used a combination of GO terms and the SynGO knowledge base on biological process ontology terms (ref. ^83^, Supplementary Table 2, Biological process tab) to highlight expression values in GO categories in our dataset. We selected all categories that included the terms ‘synapse’, ‘synaptic’, ‘dendritic’, ‘neurotransmitter’ and other key words to capture relevant categories in the SynGO knowledge base. A GO term is represented by a set of genes. We overlapped DEG detected in each cell type across time points with genes in the selected GO category, and plotted expression heatmaps for categories in which the median number of overlapping genes was more than or equal to 40 when calculated across all cell types.

#### Analysis of E14 sequencing data

E14 cells were passed through initial quality control steps using the same cut-offs as all other datasets. Cell cycle gene list was obtained from https://github.com/hbc/tinyatlas/blob/master/cell_cycle/Mus_musculus.csv and used to create cell cycle scores with Seurat’s CellCycleScoring function (Extended Data Fig. 5a). All cells were clustered using Seurat’s unsupervised clustering pipeline with FindNeighbors (dims 1:50) and FindClusters (resolution 1.5). Clusters were then split into neuron or progenitor subsets using markers for mature neurons (*Rbfox3*, *Syt1* and *Stmn2*) or progenitors (*Neurod1*, *Neurog1*, *Hes1*, *Hes5*, *Hes6*, *Emx1*, *Sox2*, *Sox9*,* Lhx2* and *Ascl1*). Neurons were mapped to cell types in the E16–P65 dataset using the label transfer method described above (‘Defining cell types’ section) (Extended Data Fig. 5c). Identity score (Extended Data Fig. 5e,f) was calculated as the value on the diagonal of Extended Data Fig. 5d, where cells were mapped to P65 cells. Progenitors were clustered using Seurat’s unsupervised clustering pipeline with FindNeighbors (dims 1:50) and FindClusters (resolution of three) (Extended Data Fig. 5h). Progenitor cells were mapped to E16 regions using the label transfer strategy described above, with the same thresholds (Extended Data Fig. 5j), although removing thresholds completely did not qualitatively change the result.

#### Developmental trajectory quantification

To calculate developmental trajectories as in Fig. 2a–c, we followed the approach described previously^28^, to calculate distance between centroids in high-dimensional principal component space. We subset each cell type across all ages, performed SCTransform to normalize and identify 2,000 highly variable genes, and performed PCA (100 principal components). We then decided how many principal components to use for each cell type by asking how many of the top principal component are needed to explain 20% of variance in the data; if more than 100 principal components were needed, we used 100 principal components. We then found the centroid at each age for each principal component in gene expression space, and calculated the Manhattan distance between each centroid and the P65 centroid. This gives us a distance value for each age relative to P65. This distance generally decreased with age, indicating maturation, but on occasions in which it increased, we set the distance value at the later age to that of the previous age. This step partly excludes transient movement away from adult (for example, the transient upregulation of gene expression programs related to axon guidance, which may be low at early and late ages but high at intermediate ages), but allowed us to better quantify how ‘adult-like’ each cell type is at each age.

We also performed nearest neighbour analysis as an alternative measure of maturity state (Extended Data Figs. 6e and 9a), following the method described previously^27^ and associated code downloaded from https://zenodo.org/records/7113422. We focused on each cell type individually and constructed *k*-nearest neighbours graphs that included all age categories, with each graph comprising 50 neighbours. To assess the maturity level for each age group, we calculated the fraction of nearest neighbours that originated from adult nuclei. This involved tallying the number of adult nuclei serving as nearest neighbours to the nuclei of a specific age. Subsequently, we normalized this total by the overall count of nearest neighbours, thereby deriving a proportion that reflects the maturity state based on the presence of adult nuclei among the nearest neighbours.

#### Signalling gene expression analysis

All gene expression quantifications plotted were performed using log-normalized counts (that is, Seurat’s RNA assay, data slot). We compiled lists of genes belonging to various signalling classes (Supplementary Table 3, based on refs. ^84,85^) and computed module scores using Seurat’s AddModuleScore function. Module scores were averaged among cells within an age and then *z*-scored across age. Refinement Score was calculated for each gene set by first summing the number of significant devDEGs (‘devDEG analysis’ section above) between P10 and P65 and then normalizing by dividing by the number of gene × cell type combinations (after excluding combinations for which no expression is found at any age).

NeuronChat analysis^35^ was performed following tutorials from https://github.com/Wei-BioMath/NeuronChat. Ligand–receptor interactions present in the hypothalamus but not in the original interaction database were added using The IUPHAR/BPS Guide to Pharmacology^85^ (Supplementary Table 4), and then all links that were not neuropeptide and monoamine links were removed. run_NeuronChat was run using *M* = 100, mean_method = ‘mean’ and FDR = 0.01. Links with ligand abundance less than 0.025 were filtered out.

#### Distance analysis to determine male–female or mutant–control differences

We adapted a distance metric analysis described previously^28^ (similar to that used in ref. ^86^ for analysis of neuronal activity data) to ask whether (1) male and female or (2) mutant and control samples cluster in distinct regions of high-dimensional PCA space, separately for each cell type. To calculate distances between sample centroids, we used procedures and parameters similar to those described above for developmental trajectory calculation. We subset each cell type and, including both male and female or mutant and control samples, performed SCTransform to normalize and detect 2,000 highly variable genes, and performed PCA (100 principal components). We then decided how many principal components to use for each cell type by asking how many of the top principal components are needed to explain 20% of variance in the data; if more than 100 principal components were needed, we used 100 principal components. Then, for each sample (for example, four male and four female samples), we quantified the sample’s centroid in principal component space. We then calculated the Manhattan distance between all pairs of sample centroids. Our effect size (‘Male-female PCA distance, normalized’ or ‘Mutant-control PCA distance, normalized’) was calculated as the average across all male-to-female or mutant-to-control distances, normalized by the average of intra-sex or intra-genotype distances. This ratio quantifies how separated genotypes or sexes are compared to separation within each group. We calculated a two-sided *t*-test *P* value between all inter-sex versus intra-sex distances, or all inter-genotype versus intra-genotype distances. In Fig. 4d and Extended Data Fig. 9b,c, any raw *P* value greater than 0.05 was considered not significant and results were validated by sexDEG analysis. For Fig. 5a and Extended Data Fig. 10a, Benjamini–Hochberg multiple comparisons corrected adjusted *P* values were used to determine which cell types were NS. For sex analysis, to increase our n number, we included control samples from mutant–control experiments. For mutant analysis, to increase our *n* number, we included C57BL/6J samples as controls at the appropriate age, and confirmed minimal detectable differences between these samples and littermate control samples. We did not collect heterozygous control littermates for *Trpm8* mice, and instead used same-age C57BL/6J samples as controls. Plots were generated using the ggplot2 R package (RRID:SCR_014601).

#### sexDEG analysis

To determine DEGs between sexes, we performed sample-pseudobulk-based DESeq2 (RRID:SCR_015687) differential expression testing for each cell type. After removing mitochondrial, ribosomal, Y chromosome and X inactivation genes, we split the dataset by ages. Then, we aggregated raw counts from cells of the same sample to use as input for DESeq2 and shrunk the log_2_ fold changes using lfcShrink (type = ‘apeglm’) to generate lists of DEGs between either male and female samples (sexDEGs). DEGs with adjusted *P* value less than 0.05 were considered significant and used for downstream analysis. To increase our *n* number, we included control samples from mutant–control experiments. For GO term analysis we used gprofiler2 in R.

#### Augur classifier analysis

To identify which cell types have a notable change in expression between the control and mutant conditions, we used the package Augur v.1.0.3 (RRID:SCR_023964)^87^, which performs cross-validated random-forest classifier analysis of single-cell datasets. First, we split the dataset by age and removed mitochondrial and ribosomal genes. Then we applied Augur (var_quantile=0.9, subsample_size=20) to generate area under the curve scores. For this analysis, control data included only control littermates with no added C57BL/6J samples (except for *Trpm8* mutants, where C57BL/6J samples were the only available controls).

#### Pseudotime analysis of *Trpc2* mutant and control datasets

For each cell type, pseudotime was calculated across all ages from C57BL/6J data using Slingshot^60^ on log-normalized gene expression data and the top 20 principal components calculated from 2,000 variable features. Then, for each mutant or control *Trpc2* sample, cells from that sample were projected into the C57BL/6J 20-PC space (using stats::predict) and Slingshot prediction was performed (using slingshot::predict) to give each cell a predicted pseudotime value. Pseudotime values were averaged across all cells within each sample, and then between samples, to give data shown in Fig. 5c. Two-sided *t*-test was performed to calculate *P* value between control and mutant cell type pseudotime values.

#### Shared neuronal maturation gene expression

To identify a set of genes that increase across age in all neuronal types, we first created a Seurat object with equal numbers of cells from each cell type by randomly selecting ten cells of each cell type at E16/E18 or at P65. Then, we used Seurat’s FindMarkers to identify genes that were more highly expressed among P65 cells than E16/E18 cells. From this list, we selected genes with average log_2_-transformed fold change greater than 0.5 that were expressed in at least 30% of P65 cells (pct.1 > 0.3). This yielded a list of 243 genes. We used Seurat’s AddModuleScore with this list of genes for the entire C57BL/6J dataset and found that the module score for these genes increased across age in every cell type (data not shown separately for each cell type; data shown altogether in Fig. 5d, left, underlying data are averaged within a cell type). We then used Seurat’s AddModuleScore with this gene set on *Trpc2*^−/+^ or *Trpc2*^−/−^ cells and averaged the values within a cell type (Fig. 5d, right).

### snATAC-seq analysis

snATAC-seq profiles were filtered to include only nuclei with at least 500 fragments, and only nuclei with paired snRNA-seq profiles passing quality control as described above. C57BL/6J samples were split into two replicates, with each replicate containing one male and one female sample at each age. Peaks were called for each cell type in each replicate using macs2 (ref. ^88^) callpeak command with parameters ‘–shift -100 –extsize 200 –nomodel –callsummits –nolambda –keep-dup all -q 0.05’. Only reproducible peaks that are present in both replicates were kept (*n* = 778,573 peaks). Finally, to compile a union peak set, we combined peaks from all cell types and extended the peak summits by 200 bp on either side. Overlapping peaks were then handled using an iterative removal procedure. First, the most significant peak, that is, the peak with the smallest *P* value, was kept and any peak that directly overlapped with it was removed. Then, this process was iterated to the next most significant peak and so on until all peaks were either kept or removed due to direct overlap with a more significant peak. Differentially accessible peaks were identified using the getMarkerFeatures() function from the ArchR^89^ package using a Wilcoxon rank sum test and accounting for bias introduced by TSSEnrichment and log_10_(nFrags). Peaks with FDR ≤ 0.1 and log_2_ fold change greater than or equal to 0.25 were considered cell type-specific. Known transcription factor binding motifs present in the ‘vierstra’ motifset were assigned to differentially accessible genomic regions and motif enrichment was evaluated using a hypergeometic test implemented by the peakAnnoEnrichment() function. Enriched motifs were those with minimum *P* adjusted enrichment of 20, top three shown per cell type (Extended Data Fig. 4b). Motif footprints were calculated by combining all peaks harbouring a given motif in aggregate and accounting for Tn5 insertion bias using the the getFootprints() function.

### Viral injections and immunohistochemistry

To visualize synaptic puncta in Gal+ or Opn5+ neurons, we injected 200 nl of AAV2/9-hSyn-FLEx-loxP-Synaptophysin-mGreenLantern-T2A-GAP43-mScarlet (provided by L. Schwarz; details of its construction can be found in ref. ^90^) at a titre of 4.9 × 10^12^ into the POA of P0 *Gal-cre* or *Opn5-cre* pups (*Gal*-*cre*: 2.15 mm anterior to lambda; 0.25 mm right of the midline; 3.9 mm deep; Opn5-Cre: 2.4 mm anterior to lambda; 0.1 mm right of the midline; 4.25 mm deep). Animals were perfused at P10 with PBS followed by 4% PFA after deep anaesthesia by isoflurane. Brains were dissected out and placed in 4% PFA for 1–2 nights, then vibratome sectioned at 60 μm. Green fluorescent protein (GFP) immunofluorescence was used to amplify Synaptophysin-mGreenLantern signal, along with VIP antibody staining. Floating coronal sections were washed three times (5 min each time) in 1× PBS with 0.3% Triton X-100 (PBST), then blocked for 1 h in Animal Free Blocker 5× Concentrate (Vector Laboratories) diluted to 1× in PBST. All washes and blocking were performed at room temperature with gentle shaking at 100 rpm. Sections were then placed in blocking solution with primary antibodies (GFP: 1:1,000 dilution, Aves Laboratories, RRID:AB_2307313; VIP: 1:1,000 dilution, Abcam) and left overnight with gentle shaking at 4 °C. Sections were again washed three times for 5 min each in PBST, then incubated in blocking solution with secondary antibodies (1:1,000 dilutions of (1) goat anti-chicken IgY, AlexaFluor 488 conjugate, Jackson ImmunoResearch, RRID:AB_2337390 and (2) goat anti-rabbit IgG, AlexaFluor 647 conjugate, Jackson ImmunoResearch, RRID:AB_2338072) for 2 h. Following three more washes as before, sections were mounted using prolong gold and kept at room temperature for at least one night before imaging. Sections were imaged at ×10 or ×40 using either a Zeiss Axio Imager.Z2 at ×40 or Zeiss LSM900 Confocal Microscope.

### RNA in situ hybridization

Double- and triple-label fluorescence in situ hybridization experiments were performed using the RNAscope Assay V2 kit (Advanced Cell Diagnostics). Brains were harvested and frozen in optimal cutting temperature medium, then sliced on the cryostat (P18+, 16 μm; P0–P10, 18 μm; E18, 14 μm). The RNAscope Assay was performed according to the manufacturer’s instructions, with one exception: For E18 brains, protease treatment was restricted to 10 min at room temperature, compared to 30 min at room temperature for all other ages. Slides were imaged on an Axio Scan.Z7 microscope at ×20.

### Behaviour assays

For all behaviour tests, litters were obtained by crossing *Trpc2*^−/+^ females with *Trpc2*^−/−^ males, and removing males after 2 weeks cohabitation, so that litters were born with only mothers present. Pups were toe-clipped at P7 for identification and genotyping for *Trpc2* and sex. All assays were blinded for both experiment and behaviour scoring, with pups split into group 1 or group 2, with random assignment of group to genotype. All assays were performed during the first 6 h of the dark cycle under dim red light.

#### Maternal retrieval

Assays were performed when pups were P9–P10. 1–2 pups of each genotype were removed from the litter and placed in a fresh cage. Then, one *Trpc2*^−/−^ and one *Trpc2*^−/+^ pup, both of the same sex, were selected and simultaneously placed in two corners of the cage opposite the nest, where the mother typically went when the cage lid was lifted. Experiments were balanced such that pups from each genotype were placed in the corner slightly closer to the nest at equal levels. Assays were video recorded and retrieval latency was manually scored (blinded to genotype) for each pup as the time for the mother to grab a pup, return it to the nest and release it from her mouth (Extended Data Fig. 10g).

#### Maternal approach

Assays were performed when pups were P13–P14, following a 30-min isolation period at room temperature (described below, Isolation vocalizations). Mothers were anaesthetized with ketamine–xylazine mix (10 mg ml^−1^ ketamine and 1 mg ml^−1^ xylazine) and placed on her side in one corner of the cage. A pup was then introduced 15 cm away from the mother’s abdomen, and facing the mother. Time to first contact between pup and any part of the mother was scored manually (blinded to genotype) (Extended Data Fig. 10h).

#### Littermate approach and behavioural quiescence

Assays were performed when pups were P11–P12. Two to four pups, all of the same genotype, were placed in a fresh cage, one by one, at 3-min intervals, at 10 cm apart from one another. Typically, pups of this age will spend a brief time moving around the cage, before entering a prolonged period of behavioural quiescence. When another pup is added to the cage, pups often encounter one another, huddle together and stop moving. We manually scored (blinded to genotype) both time to first contact of a littermate (Extended Data Fig. 10i) and time to enter behavioural quiescence (Extended Data Fig. 10j).

#### Isolation vocalizations

P13–P14 pups were placed individually in fresh cages and recorded with ultrasonic microphones (u256, Batsound) embedded in cage lids, for 30 min. We used the AVA package^91^ (https://autoencoded-vocal-analysis.readthedocs.io/en/latest/index.html) along with custom Python scripts to perform amplitude-based ultrasonic vocalization segmentation. On P14, pups were tested with a heating pad (Small Animal Heated Pad, K&H) underneath the cage, with all cage bottom material removed. Infrared gun temperature measurements of the cage floor consistently showed 33.3–36.7 °C (heating pad) or 21.7–23.9 °C.

### Statistics and reproducibility

Data were processed and analysed using a combination of R and Python codes. Sample sizes were chosen based on common practice in single-nucleus sequencing experiments. Individual data points were plotted wherever possible. Boxplots represent the median, first and third quartiles (hinges) and 1.5× interquartile range (whiskers). Outliers are shown wherever individual data points are not plotted. All data were analysed using two-tailed non-parametric tests. *P < 0.05, **P < 0.001 and ***P < 0.0001. Statistical details are given in the respective figure legends.

### Reporting summary

Further information on research design is available in the Nature Portfolio Reporting Summary linked to this article.

## Online content

Any methods, additional references, Nature Portfolio reporting summaries, source data, extended data, supplementary information, acknowledgements, peer review information; details of author contributions and competing interests; and statements of data and code availability are available at 10.1038/s41586-025-08603-0.

## Supplementary information

Supplementary Discussion This section includes further discussion related to E14 progenitor analysis, feeding and sleep/wake related genes and sex differences.

Reporting Summary

Supplementary Table 1 Cell type mapping to previous datasets. Correspondence between cell types identified here and those identified in Moffitt et al. and previous functional studies.

Supplementary Table 2 Transcriptomic groups and associated brain regions. This table lists the excitatory and inhibitory subregional groups shown in Fig 1c and the associated POA regions that are represented within each group.

Supplementary Table 3 Signalling gene lists. This table lists different categories of genes analysed in Fig. 3.

Supplementary Table 4 Neuropeptide links added to NeuronChat database. This table lists neuropeptide-receptor pairs that were added to the NeuronChat database for the analysis in Fig 3.

## Data Availability

All sequencing data generated in this study have been deposited in the Gene Expression Omnibus (GEO) under accession GSE280964.

## Code availability

Code used for analyses in this study are available at https://github.com/harriskap/POA-development.
