# Supplementary material for: Sensory input, sex and function shape hypothalamic cell type development
Source: Nature. 2025 Mar 5;647(8088):157–68. doi: 10.1038/s41586-025-08603-0 (PMC12589138; doi:10.1038/s41586-025-08603-0)
Supplement: Supplementary file 1 — This section includes further discussion related to E14 progenitor analysis, feeding and sleep/wake related genes and sex differences. [file 41586_2025_8603_MOESM1_ESM.pdf]

nature portfolio

https://doi.org/10.1038/s41586-025-08603-0

Supplementary information

Sensory input, sex and function shape hypothalamic cell type development

In the format provided by the authors and unedited

Supplementary Discussion

Neurogenesis and diversification at E14

Our E14 data show a lack of regional and cell type identity in progenitors (Ext Fig 5j-k), while newborn neurons at the same age, which were born within the past 1-2 days, are diversified (Ext Fig 5d). How does rapid diversification of newborn POA neurons occur? In one scenario, a rapid engagement of gene regulatory networks upon neurogenesis establishes generic neuronal identity as well as gene expression patterns, including e.g. receptors rendering cells sensitive to local morphogen cues, which may lead to region-specific gene expression patterns. Alternatively, progenitors may already display subtle region- and cell-type-specific gene expression differences that are undetectable by our relatively shallow sequencing approach. Another possibility is that progenitors have primed enhancer elements, which are poised to be triggered upon neurogenesis; analysis of E14 snATAC-seq or other epigenetic data could reveal such a mechanism, in which identity is encoded at the level of chromatin but not gene expression. Altogether, it appears that cell type identity diversification is tied to neurogenesis in the POA, unlike other brain regions[26,62-64]. Finally, we sought to confirm the expression of key progenitor marker genes in the embryonic POA using the Allen Developing Brain ISH Atlas, and found that genes such as Hes5 are indeed spatially localized to the embryonic POA.

Feeding & metabolism regulation

The development of feeding and metabolism regulation undergoes several major transitions through birth and weaning, in which new regulatory loops are progressively added, such as arcuate nucleus control[106]. The POA receives significant input from Agrp+ and Pomc+ neurons in the arcuate nucleus[105]. Arcuate neurons are regulated by circulating leptin and ghrelin through their cognate receptors Lepr and Ghsr and they exert their function in controlling food intake partly through release of neuropeptides that bind to the receptor Mc4r[106]. Mc4r expression is detectable in seven POA cell types, including e-M1:MPN/Pe$^{Mc4r/C1ql2}$, which controls social drive in female mice, and e-F1:PeFA^{Ucn3}, which regulate infanticide. In all seven cell types, Mc4r expression increased at P10-18 (Fig 3c and Extended Data Fig 7g). The leptin receptor Lepr is also expressed in e-M1:MPN/Pe$^{Mc4r/C1ql2}$, as well as in e-A1:AvPe/MnPO$^{Brs3/Vglut2}$, which has reported roles in parenting behavior and body temperature regulation. Lepr showed expression increases in these and other cell types with a similar timing as Mc4r (Extended Data Fig 7h). This suggests that peripheral (leptin) and Arcuate regulation of POA cell types, including cell types involved in social behavior, may begin between P4 and P10. This timing coincides with both the maturation of Arcuate projections[106] and a surge in circulating leptin, shown to be critical for the establishment of Arcuate neuronal circuitry[107]. Prior to the development of Arcuate projections, the brainstem is a prominent regulator of feeding behavior, through projections to PVN already present at birth[106]. In adults, Glp1 signaling from brainstem to Glp1r+ neurons in PVN regulates food intake[108]. Consistent with an earlier regulation of feeding by brainstem compared to

Arcuate nucleus, we find Glp1r expression in PVN is already high at P4 (Fig 3d). Finally, expression of the ghrelin receptor Ghsr was enriched in two POA cell types involved in mating behavior, including Kiss1+ neurons (Fig 3e). Ghsr expression increased particularly late in these two cell types, between P18 and P65. Altogether, these data indicate three developmental phases of metabolic control on POA cell types, marked by the onset of distinct receptor expression patterns: (1) brainstem regulation of PVN via Glp1 at P4; (2) Arcuate and peripheral (leptin) regulation of various POA cell types, including cell types involved in thermoregulation and social behavior, at P10; (3) ghrelin regulation of periventricular cell types involved in social behavior at P28. We validated the cell-type-specific gene expression dynamics of Ghsr, Glp1r, and Lepr using RNA in situ hybridization (Extended Data Fig 8e-g).

Sleep/wake and dopaminergic regulation

Young animals show major changes in sleep and circadian rhythms, from early postnatal life and extending well into adolescence. We therefore examined developmental changes in orexin/hypocretin and histamine signaling. The histamine receptor Hrh3 showed no detectable expression until P4, followed by large increases from P10 to P28 (Fig 3f). These changes occurred in neurons in the HDB, part of the basal forebrain, a key brain region in arousal control. Unexpectedly, Hrh3 was also enriched in MPN neurons with roles in social drive and mating and showed similar changes with age, suggesting dynamic regulation of social behavior by sleep/wake signaling systems (Fig 3f). Such regulation was also apparent in hypocretin receptor expression: Hcrtr1 and Hcrtr2 were expressed in MPN neurons with well-established roles in parenting and social drive, as well as basal forebrain neurons with known roles in sleep and arousal, including cholinergic neurons (Fig 3g-h; Extended Data Fig 7i-j). However, unlike Hrh3, Hcrtr1 and Hcrtr2 expression showed distinct developmental dynamics in cell types involved in social behavior (increased expression at P18-P65 (Fig 3h)) compared to cell types involved in sleep/wake control (a surprisingly transient peak of expression at P4-P10 (Fig 3g and Extended Data Fig 7i-j)). Hypocretin signaling may therefore play distinct roles, via different sets of cell types, at P4-P10 versus later ages. Like metabolic signals, sleep/wake signals are poised to potentially affect social behavior, via histamine and hypocretin signaling in MPN cell types particularly later in life.

Dopamine signaling in the hypothalamus is best studied for its role in social behavior[47,109]. Dopaminergic neurons in various brain regions are also involved in sleep/wake control[110]. Consistent with both roles, we found dopamine receptor expression enriched in sub-regions involved in social behavior (BNST, MPN) and sleep/wake control (basal forebrain, SCN) (Extended Data Fig 7k). Dopamine receptors exhibited complex receptor- and cell-type-specific developmental dynamics in BNST and MPN cell types (Extended Data Fig 7l), including expression as early as E16. We identified two related cell types that express Th, Ddc, and Slc18a2 and are thus poised to synthesize, package, and release dopamine, and which play key roles in mating and parenting behavior[1,47,109]; expression of these genes was present at low levels at E16 and increased with age (Extended Data Fig 7m). Dopamine receptor expression in basal forebrain and SCN showed region-specific differences in developmental dynamics. Drd1 and Drd3 were both expressed in basal forebrain cell types involved in sleep/wake regulation, but only from P10 onwards (Fig 3i and Extended Data Fig 7l). In contrast, Drd1 expression in SCN cell types that dictate circadian rhythms was present as early as E18 and constant through to adulthood (Fig 3j). We validated sub-region-specific gene expression dynamics of Hrh3 and Drd1 using RNA in situ hybridization (Extended Data Fig 8h-j). In summary, dopamine receptor

expression shows highly region- and cell-type-specific developmental dynamics, suggesting complex regulation of monoamine signaling across age.

Sex hormone signaling

Sex hormone signaling drives sexual differentiation in the POA both at puberty (P28 to P65) and perinatally (E18-P4), when a transient male-specific surge in testosterone impacts gene expression following conversion to estrogen by the enzyme aromatase[43,111]. Consistent with this model, the estrogen receptor Esr1 was enriched perinatally in MPN/Pe and BNST, two regions with well-established sex differences in gene expression[43,45] (representative example populations shown in Fig 3k-o; others shown in Extended Data Fig 7o-r). Esr1 expression was largely maintained in those cell types through adulthood and showed small increases in additional cell types (Fig 3k and Extended Data Fig 7o). Estrogen signaling in the male brain has been proposed to occur in a paracrine manner: testosterone is converted to estrogen in a small number of aromatase-expressing cell types, followed by estrogen binding to Esr1 in a larger number of Esr1-expressing cell types[1]. Indeed, we observed aromatase expression at birth in three cell types: i-B1:BNST$^{Aro/Cdh24}$, i-M2:Pe/SHy$^{Gal/Th}$, and i-M3:Pe/VMPO$^{Kiss1/Th}$ (Fig 3l). Intriguingly, all three showed a decrease in aromatase expression with age, whereas a fourth cell type, i-B6:BNST$^{Aro/Tac1}$, showed an age-dependent increase in aromatase expression (Fig 3l and Extended Data Fig 7n). This suggests that distinct sets of neurons convert testosterone to estrogen at birth compared to adulthood. Further, i-M2:Pe/SHy$^{Gal/Th}$ and i-B6:BNST$^{Aro/Tac1}$ did not express detectable levels of Esr1, consistent with paracrine estrogen signaling. In contrast to Esr1, expression of the testosterone receptor Ar, the progesterone receptor Pgr, and the prolactin receptor Prlr is low perinatally and showed substantial increases with age, in a larger number of cell types (Fig 3m-o and Extended Data Fig 7p-r). Consistent with a role in physiological changes during pregnancy[56], Prlr was expressed in cell types involved in homeostatic control functions, including fever, sleep, and thermoregulation (Fig 3o and Extended Data Fig 7r). We validated the up-regulation of Ar from P0 to P10 to P65 using RNA in situ hybridization (Extended Data Fig 8k).

Sex differences in specific cell types of interest

The two cell types that showed the highest degree of sex difference were e-M1:MPN/Pe$^{Mc4r/C1ql2}$ and i-M5:MPN/StHy$^{Gal/Fbn2}$, both of which were recently shown to play sex-specific roles in social behavior[1,6]. A gene ontology analysis of i-M5:MPN/StHy$^{Gal/Fbn2}$ sexDEGs indicated age-specific enrichment in several neuronal and developmental processes (Extended Data Fig 9f). SexDEGs unique to P4-P10 were enriched in genes related to nervous system development and synapse assembly. SexDEGs unique to P18-P28 were enriched in genes involved in synaptic transmission, as well as axon development and guidance. At P65, sexDEGs were no longer enriched in genes relating to developmental processes but were instead enriched in genes related to neuropeptide and hormone signaling. In e-M1:MPN/Pe$^{Mc4r/C1ql2}$, a similar progression from sexDEGs related to neurodevelopment early in life to specific neuronal functions later in life was observed, albeit with distinct functions such as GABAergic synapses or Trkb and protein kinase C signaling (Extended Data Fig 9e-f). These data indicate that while the perinatal period is the key stage for establishing sex differences in these two cell types, sex-specific expression changes continue at later ages in a cell-type specific manner to affect neuronal development and function.

To determine how sex hormone signaling might underlie dynamic sex differences in gene expression, we examined Esr1 motifs in our chromatin accessibility dataset. Transcription factor footprinting analysis supported higher accessibility at Esr1 motifs in males compared to females in i-M5:MPN/StHy$^{Gal/Fbn2}$ neurons at P0 and P10, but not at P18 (Extended Data Fig 9j). This suggests that the perinatal testosterone surge may exert sex-specific effects via Esr1 through P10 (to be confirmed by future chromatin immunoprecipitation sequencing experiments), but that Esr1 function is no longer sex-specific by P18, which may partly explain dynamic changes in gene expression across age (Extended Data Fig 9j). While most of the sexDEGs were identified after the P0 testosterone surge, a small number of sexDEGs were apparent prior to P0, such as Pdzrn4 (Extended Data Fig 9h), which we confirmed by RNA in situ hybridization (Extended Data Fig 9i).

The cell type i-M3:Pe/VMPO$^{Kiss1/Th}$ was the only population to show a replicable sex difference in cell number (Extended Data Fig 9k), with a 3:1 female bias as previously reported[1,47]. Sex differences in transcriptomic cell type number have also been reported in BNST[1,44], which was not apparent in our dataset, perhaps due to variability in BNST dissection. Differences in BNST cell number arise perinatally via estrogen-dependent protection from apoptosis in males but not females[45]. In contrast, how and when female-biased cell number differences emerge in i-M3:Pe/VMPO$^{Kiss1/Th}$ is not known. Our data indicate that, similar to BNST cell types, the sex bias in cell number in POA emerges perinatally (Extended Data Fig 9k). While i-M3:Pe/VMPO$^{Kiss1/Th}$ showed numerous sexDEGs before and after birth (Fig 4d-e, Extended Data Fig 9b), we did not observe sex differences in genes related to apoptotic pathways. Finally, although microglia and prostaglandin signaling have been implicated in the emergence of perinatal sex differences[113], low cell numbers prevented us from assessing sex differences in these cells, and we did not find sexDEGs associated with prostaglandin signaling.

105. Yang, S. *et al.* An mPOA-ARCAgRP pathway modulates cold-evoked eating behavior. *Cell Reports* **36**, 109502 (2021).

106. Zeltser, L. M. Feeding circuit development and early-life influences on future feeding behaviour. *Nature Reviews Neuroscience* **19**, 302–316 (2018).

107. Bouret, S. G., Draper, S. J. & Simerly, R. B. Trophic Action of Leptin on Hypothalamic Neurons That Regulate Feeding. *Science* **304**, 108–110 (2004).

108. Liu, J. *et al.* Enhanced AMPA Receptor Trafficking Mediates the Anorexigenic Effect of Endogenous Glucagon-like Peptide-1 in the Paraventricular Hypothalamus. *Neuron* **96**, 897-909.e5 (2017).

109. Zhang, S. X. *et al.* Hypothalamic dopamine neurons motivate mating through persistent cAMP signalling. *Nature* **597**, 245–249 (2021).

110. Adamantidis, A. R. & Lecea, L. de. Sleep and the hypothalamus. *Science* **382**, 405–412 (2023).

111. Wu, M. V. & Shah, N. M. Control of masculinization of the brain and behavior. *Current Opinion in Neurobiology* **21**, 116–123 (2011).

112. Ladyman, S. R., Carter, K. M., Gillett, M. L., Aung, Z. K. & Grattan, D. R. A reduction in voluntary physical activity in early pregnancy in mice is mediated by prolactin. *Elife* **10**, e62260 (2021).

113. Lenz, K. M., Nugent, B. M., Haliyur, R. & McCarthy, M. M. Microglia Are Essential to Masculinization of Brain and Behavior. *J. Neurosci.* **33**, 2761–2772 (2013).
