# Supplementary material for: Sensory input, sex and function shape hypothalamic cell type development
Source: Nature. 2025 Mar 5;647(8088):157–68. doi: 10.1038/s41586-025-08603-0 (PMC12589138; doi:10.1038/s41586-025-08603-0)
Supplement: Supplementary file 2 — Reporting Summary [file 41586_2025_8603_MOESM2_ESM.pdf]

nature portfolio

Corresponding author(s): Catherine Dulac

Last updated by author(s): Oct 9, 2024

Reporting Summary

Nature Portfolio wishes to improve the reproducibility of the work that we publish. This form provides structure for consistency and transparency in reporting. For further information on Nature Portfolio policies, see our Editorial Policies and the Editorial Policy Checklist.

Statistics

For all statistical analyses, confirm that the following items are present in the figure legend, table legend, main text, or Methods section.

n/a	Confirmed	
☐	☒	The exact sample size (*n*) for each experimental group/condition, given as a discrete number and unit of measurement
☐	☒	A statement on whether measurements were taken from distinct samples or whether the same sample was measured repeatedly
☐	☒	The statistical test(s) used AND whether they are one- or two-sided<br>*Only common tests should be described solely by name; describe more complex techniques in the Methods section.*
☐	☒	A description of all covariates tested
☐	☒	A description of any assumptions or corrections, such as tests of normality and adjustment for multiple comparisons
☐	☒	A full description of the statistical parameters including central tendency (e.g. means) or other basic estimates (e.g. regression coefficient) AND variation (e.g. standard deviation) or associated estimates of uncertainty (e.g. confidence intervals)
☐	☒	For null hypothesis testing, the test statistic (e.g. *F*, *t*, *r*) with confidence intervals, effect sizes, degrees of freedom and *P* value noted<br>*Give P values as exact values whenever suitable.*
☒	☐	For Bayesian analysis, information on the choice of priors and Markov chain Monte Carlo settings
☒	☐	For hierarchical and complex designs, identification of the appropriate level for tests and full reporting of outcomes
☐	☒	Estimates of effect sizes (e.g. Cohen's *d*, Pearson's *r*), indicating how they were calculated

Our web collection on statistics for biologists contains articles on many of the points above.

Software and code

Policy information about availability of computer code

Data collection	Single-nucleus multiome-seq: Raw sequencing data was processed using the CellRanger Arc (v2.0.1) pipeline with reference genome mm10. Default parameters were used for read alignment, uniqe fragment or transcript counting, and filtering of high-quality nuclei.
Data analysis	The following packages were used to analyze and visualize the data: ComplexHeatmap (v2.18.0), dendextended (v1.17.1), NeuronChat (v1.0.0), Seurat (v4.4.0), pheatmap (v1.0.12), ggplot2 (v.3.4.4), gprofiler2 (v0.2.2), edgeR (v3.32.1), DESeq2 (v1.30.1), ArchR (v1.0.1), Augur (v1.0.3), slingshot (v2.10.0), python (v3.10.9), AVA (v0.3.1).<br>Custom code used in this study is available at https://github.com/harriskap/POA-development.

For manuscripts utilizing custom algorithms or software that are central to the research but not yet described in published literature, software must be made available to editors and reviewers. We strongly encourage code deposition in a community repository (e.g. GitHub). See the Nature Portfolio guidelines for submitting code & software for further information.

Data

Policy information about availability of data

All manuscripts must include a data availability statement. This statement should provide the following information, where applicable:

- Accession codes, unique identifiers, or web links for publicly available datasets
- A description of any restrictions on data availability
- For clinical datasets or third party data, please ensure that the statement adheres to our policy

The data generated by this study are in GEO (GSE280964).

Research involving human participants, their data, or biological material

Policy information about studies with human participants or human data. See also policy information about sex, gender (identity/presentation), and sexual orientation and race, ethnicity and racism.

Reporting on sex and gender	N/A
Reporting on race, ethnicity, or other socially relevant groupings	N/A
Population characteristics	N/A
Recruitment	N/A
Ethics oversight	N/A

Note that full information on the approval of the study protocol must also be provided in the manuscript.

Field-specific reporting

Please select the one below that is the best fit for your research. If you are not sure, read the appropriate sections before making your selection.

☒ Life sciences ☐ Behavioural & social sciences ☐ Ecological, evolutionary & environmental sciences

For a reference copy of the document with all sections, see nature.com/documents/nr-reporting-summary-flat.pdf

Life sciences study design

All studies must disclose on these points even when the disclosure is negative.

Sample size	No statistical methods were used to predetermine sample sizes, which were chosen to be consistent with standard practices in the field (Moffitt et al., 2018; Osterhout et al., 2022; Gegenhuber et al., 2022; Cheng et al., 2023).
Data exclusions	No data were excluded.
Replication	All experiments were repeated at least twice per genotype, age, and sex combination. For each experimental sample, brain tissue from 2-3 animals was pooled in order to reduce dissection variability. All attempts at replication were successful.
Randomization	Randomization was not performed in this study, because no manipulations were performed in which animals would be assigned to experimental groups.
Blinding	All behavior assays were performed and quantified by researchers blind to the genotypes of the animals. Blinding was not performed for snRNA-seq and snATAC-seq analysis, because knowledge of sample identity is required to perform data analysis and visualize the results. RNA in situ hybridization experiments were performed by a researcher blind to the sex and genotype of the animals, but not the age, which was not possible due to brain size differences.

Reporting for specific materials, systems and methods

We require information from authors about some types of materials, experimental systems and methods used in many studies. Here, indicate whether each material, system or method listed is relevant to your study. If you are not sure if a list item applies to your research, read the appropriate section before selecting a response.

Materials & experimental systems

n/a	Involved in the study	
☐	☒	Antibodies
☒	☐	Eukaryotic cell lines
☒	☐	Palaeontology and archaeology
☐	☒	Animals and other organisms
☒	☐	Clinical data
☒	☐	Dual use research of concern
☒	☐	Plants

Methods

n/a	Involved in the study	
☒	☐	ChIP-seq
☒	☐	Flow cytometry
☒	☐	MRI-based neuroimaging

Antibodies

Antibodies used

GFP, Aves Labs, Davis, CA
VIP, Abcam, Waltham, MA
goat anti-chicken IgY, AlexaFluor 488 conjugate, Jackson ImmunoResearch, West Grove, PA
goat anti-rabbit IgG, AlexaFluor 647 conjugate, Jackson ImmunoResearch, West Grove, PA

Validation

GFP:
Manufacturer quality control notes:
"Antibodies were analyzed by western blot analysis (1:5000 dilution) and immunohistochemistry (1:500 dilution) using transgenic mice expressing the GFP gene product. Western blots were performed using BlokHen® (Aves Labs) as the blocking reagent, and HRP-labeled goat anti-chicken antibodies (Aves Labs, Cat. #H-1004) as the detection reagent. Immunohistochemistry used tetramethyl rhodamine-labeled anti-chicken IgY."
2297 citations listed at https://www.antibodiesinc.com/products/anti-green-fluorescent-protein-antibody-gfp

VIP:
23 citations listed at https://www.abcam.com/en-us/products/primary-antibodies/vip-antibody-ab22736#

Animals and other research organisms

Policy information about studies involving animals; ARRIVE guidelines recommended for reporting animal research, and Sex and Gender in Research

Laboratory animals

Experiments were performed on male and female mice at ages detailed in the text and figures, including E14, E16, E18, P0, P4, P9-14, P18, P28, and P65. Mutant and transgenic strains used in this study were previously published and include (with Jackson Laboratories numbers where applicable): Trpc2 knockout mice ref. 64 (021208); Cnga2 knockout mice ref. 70; Trpm8 knockout mice ref. 84 (008198); Piezo2fl ref. 85 (027720); Piezo2- ref. 86; Cdx2-Cre ref. 87 (009350); Advillin-Cre ref. 88; Gabrb3fl ref. 89.

Wild animals

This study did not involve wild animals.

Reporting on sex

All experiments were performed in both male and female mice, to examine sex differences.

Field-collected samples

This study did not involve field-collected samples.

Ethics oversight

All experiments were performed in accordance with NIH guidelines and approved by the Harvard University Institutional Animal Care and Use Committee (IACUC).

Note that full information on the approval of the study protocol must also be provided in the manuscript.

Plants

Seed stocks

Report on the source of all seed stocks or other plant material used. If applicable, state the seed stock centre and catalogue number. If plant specimens were collected from the field, describe the collection location, date and sampling procedures.

Novel plant genotypes

Describe the methods by which all novel plant genotypes were produced. This includes those generated by transgenic approaches, gene editing, chemical/radiation-based mutagenesis and hybridization. For transgenic lines, describe the transformation method, the number of independent lines analyzed and the generation upon which experiments were performed. For gene-edited lines, describe the editor used, the endogenous sequence targeted for editing, the targeting guide RNA sequence (if applicable) and how the editor was applied.

Authentication

Describe any authentication procedures for each seed stock used or novel genotype generated. Describe any experiments used to assess the effect of a mutation and, where applicable, how potential secondary effects (e.g. second site T-DNA insertions, mosiacism, off-target gene editing) were examined.
